# The *de novo* assembly and characterization of the complete mitochondrial genome of bottle gourd (*Lagenaria siceraria*) reveals the presence of homologous conformations produced by repeat-mediated recombination

**DOI:** 10.3389/fpls.2024.1416913

**Published:** 2024-08-12

**Authors:** Nannan Qin, Shanjie Yang, Yunan Wang, Hui Cheng, Yang Gao, Xiaojing Cheng, Sen Li

**Affiliations:** ^1^ College of Horticulture, Shanxi Agricultural University, Jinzhong, China; ^2^ Department of Development Planning & Cooperation, Shanxi Agricultural University, Taiyuan, China; ^3^ Department of Scientific Research Management, Shanxi Agricultural University, Taiyuan, China

**Keywords:** *Lagenaria siceraria*, mitochondrial DNA sequencing, tandem repeats, phylogenetic analysis, RNA editing site

## Abstract

**Introduction:**

Bottle gourd is an annual herbaceous plant that not only has high nutritional value and many medicinal applications but is also used as a rootstock for the grafting of cucurbit crops such as watermelon, cucumber and melon. Organellar genomes provide valuable resources for genetic breeding.

**Methods:**

A hybrid strategy with Illumina and Oxford Nanopore Technology sequencing data was used to assemble bottle gourd mitochondrial and chloroplast genomes.

**Results:**

The length of the bottle gourd mitochondrial genome was 357547 bp, and that of the chloroplast genome was 157121 bp. These genomes had 27 homologous fragments, accounting for 6.50% of the total length of the bottle gourd mitochondrial genome. In the mitochondrial genome, 101 simple sequence repeats (SSRs) and 10 tandem repeats were identified. Moreover, 1 pair of repeats was shown to mediate homologous recombination into 1 major conformation and 1 minor conformation. The existence of these conformations was verified via PCR amplification and Sanger sequencing. Evolutionary analysis revealed that the mitochondrial genome sequence of bottle gourd was highly conserved. Furthermore, collinearity analysis revealed many rearrangements between the homologous fragments of *Cucurbita* and its relatives. The Ka/Ks values for most genes were between 0.3~0.9, which means that most of the genes in the bottle gourd mitochondrial genome are under purifying selection. We also identified a total of 589 potential RNA editing sites on 38 mitochondrial protein-coding genes (PCGs) on the basis of long noncoding RNA (lncRNA)-seq data. The RNA editing sites of *nad1*-2*, nad4L*-2*, atp6*-718*, atp9*-223 *and rps10*-391 were successfully verified via PCR amplification and Sanger sequencing.

**Conclusion:**

In conclusion, we assembled and annotated bottle gourd mitochondrial and chloroplast genomes to provide a theoretical basis for similar organelle genomic studies.

## Background

Bottle gourd (*Lagenaria siceraria*) is an annual herbaceous plant in the Cucurbitaceae family that is native to Africa and cultivated worldwide (Konan et al., 2020). In addition to being an important agricultural crop, bottle gourd also has a wide range of applications in traditional medicine and crafts (Zahoor et al., 2021). Bottle gourd has high nutritional value and medicinal properties and is often used as an important rootstock for the grafting of cucurbit crops such as watermelon, cucumber and melon (Yang et al., 2013; Liang et al., 2022). Moreover, this gourd species has good low-temperature tolerance, a high affinity for grafting, and resilience in the quality of the melons it produces; furthermore, there are few obstacles to its continuous cropping, and it is highly disease- and pest-resistant (Xu et al., 2023).

As cellular organelles, mitochondria are essential for maintaining the cellular energy supply and for many biological functions (Liberatore et al., 2016; Møller et al., 2021). Mitochondria have their own genetic material, known as mitochondrial DNA (mtDNA), and their genomes contain key information that encodes proteins within the mitochondria (Huang et al., 2020). The plant mitochondrial genome is the largest known organellar genome after the nuclear genome and has unique characteristics, such as the lowest known rate of synonymous substitutions, relatively rich rearrangements, and high levels of inversion and recombination (Gualberto et al., 2014; Garcia et al., 2021; Yu et al., 2022). The mitochondrial genome harbors genes encoding key proteins in the mitochondria that are involved in the function of the cellular respiratory chain (Cho et al., 2023; Li et al., 2023). Several plant organelle RNA recognition (PORR) members, including WTF1, WTF9 and LEFKOTHEA, play a role in the splicing of introns in angiosperm organelles. *Arabidopsis thaliana* root primordium defective 1 (*rpd1*) plays a role in the splicing of introns located in the coding regions of various complex I (CI) subunits (i.e., *nad2*, *nad4*, *nad5* and *nad7*), as well as in the maturation of ribosomal *rps3* in mitochondria. Changes in the growth and development phenotypes of *rpd1* mutants and changes in respiratory activity are closely related to defects in the respiratory chain caused by CI (Edris et al., 2024). Additionally, genes in the mitochondrial genome are considered important controllers of other biological processes, including apoptosis and cell signaling (Yang H. et al., 2023). The Bcl-2 protein family regulates apoptosis by controlling mitochondrial permeability. The antiapoptotic proteins Bcl-2 and Bcl-xL reside in the outer mitochondrial membrane and inhibit cytochrome c release. Notably, the construction of recombinant fluorescent protein markers confirmed that *cox-8* is closely related to mitochondrial apoptotic cells (Shao et al., 2021).

Several studies conducted in recent years have suggested that the mitochondrial and chloroplast genomes are similar in terms of their phylogeny, biological classification, and kinship. In particular, a small number of species transmit mitochondrial DNA through paternal lines, which provides a unique explanation for the phylogenetic relationships among species from the perspective of paternal inheritance (Del Valle-Echevarria et al., 2016; Mandel et al., 2020). The paternal inheritance of mitochondria in plants was first discovered in green algae, and then, via techniques such as cytology and molecular genetics, it was found that mitochondria in cucumber and melon also exhibit paternal inheritance (Boynton et al., 1987). Mitochondria are also paternally inherited in a few other higher plant species, such as banana and kiwifruit (Faure et al., 1994; Chat et al., 2004). However, owing to the small number of seed plant species that exhibit this type of paternal inheritance, there are very few studies on the mechanism of mitochondrial paternal inheritance. In Cucurbitaceae crops, only a Psm locus on the nuclear genome has been found to control the paternal inheritance of cucumber, and after the publication of the cucumber genome, this locus was found to be located on chromosome 3 and is considered a major QTL (Al-Faifi et al., 2008). Patchy greening or necrosis of cucumber leaves and fruits is a paternal phenotype that is accompanied by a decrease in plant vigor and fertility and promotes the expression of resistance-related genes, and research has shown that this trait is regulated by the mitochondrial genes *nad5* and *atp4* (Del Valle-Echevarria et al., 2015). In addition, the phenotypes of F_1_ plants produced via positive and negative crosses between cold-resistant (CH1) and non-cold-resistant (CH4) cucumber cultivars were consistent, and the F_2_ generations from CH4×CH1 were more cold resistant than the F_2_ generations from the CH1×CH4 cross, suggesting that the mitochondrial genome may be involved in the regulation of this trait (Ali et al., 2014).

To date, the mitochondrial genomes of eight Cucurbitaceae species, including cucumber, watermelon, bitter bottle gourd and zucchini, have been sequenced, and these data lay the foundation for further elucidation of various scientific topics, such as the evolution and inheritance of the mitochondrial genome of Cucurbitaceae plants (Hansen et al., 2007; Cui et al., 2021; Niu et al., 2023; Zhou et al., 2023). Cucumber, watermelon and *Cucurbita pepo* all had 37 protein-coding genes, but the protein-coding genes among cucumber, watermelon and *Cucurbita pepo* were not identical; for example, *rps19* is found in watermelon and *Cucurbita pepo* but not in cucumber. However, *rpl10* is found in cucumber but not in watermelon or *Cucurbita pepo*. Melon has only 36 genes encoding proteins and has lost *rpl10* and *rps19* (Hansen et al., 2007; Cui et al., 2021; Niu et al., 2023; Zhou et al., 2023). Assembling the bottle gourd mitochondrial genome is extremely important for obtaining a thorough understanding of the structure, function, and evolution of bottle gourd mitochondria, but pertinent research on the mitochondrial genome of this gourd has not been performed. Thus, in this study, the bottle gourd mitochondrial genome was sequenced, assembled, and annotated, and its traits, including structural traits, were investigated. PCR assays were subsequently conducted to test the mitochondrial genomic substructure. The bottle gourd chloroplast genes were assembled via the same dataset, and homologous fragments of the bottle gourd mitochondrial and chloroplast genomes were revealed. Mitochondrial RNA editing events and their evolutionary relationships were also analyzed. In conclusion, we thoroughly analyzed the bottle gourd mitochondrial genome to provide a more complete understanding of the genome and crucial data for studying the evolution and phylogeny of bottle gourd and for informing practical applications of these data and further genetic advancements.

## Materials and methods

### Plant material

The experiment was performed in Zhuangziying village, Xugou town, Qingxu County, Taiyuan city, Shanxi Province, China. We used the bottle gourd cultivar Xiao Shounian (the leaves are small, the branching is strong, the number of fruits per plant is generally 80-100, and the fruits are approximately 3-4 cm tall and divided into upper and lower chambers) as test materials; we selected mature, plump and complete bottle gourd seeds soaked in a 55°C warm water solution and sowed them in a 32-well dish prior to cultivating them under natural light in a solar greenhouse. The whole growth period was managed according to field conventions, and no species of the same family were cultivated around the planting area. On June 13, 2023, the 10th–12th young leaves were removed from the plants by cutting them along the base of the petiole with alcohol-disinfected scissors; the leaves were rapidly placed into a precooled sampling tube, stored in liquid nitrogen, and subsequently stored in a -80°C freezer.

### DNA library construction and sequencing

The bottle gourd DNA was extracted via a plant DNA extraction kit (Tiangen, Beijing, China), and its purity, concentration, and integrity were checked via a Nanodrop instrument, Qubit quantification, and 0.35% agarose gel electrophoresis. If the DNA sample was of sufficient quality, it was transported on dry ice to Beijing Biomics Biotech Co., Ltd. (Beijing, China) for sequencing. The DNA was randomly sheared via a Covaris ultrasonic crusher, and terminal repair, A-tail addition, adapter addition, fragment screening, PCR amplification, and purification were then performed to generate the final DNA library (Meyer and Kircher, 2010). The Illumina NovaSeq 6000 high-throughput sequencing platform was used for next-generation sequencing. The raw data output was 6.63 Gb, and the filtered clean data output was 6.61 Gb. gTUBE was used to break the genomic DNA to an average length of approximately 8 kb, and DNA damage repair, end repair, linkage and Qubit library quantification were conducted. Oxford Nanopore platform sequencing was used for third-generation sequencing, and a total of 7.23 Gb of raw data was measured (Lu et al., 2016). After the connectors, short fragments (less than 500 bp in length) and low-quality data were filtered out, a total of 7.14 Gb of clean data was obtained.

### Bottle gourd mitochondrial gene splicing, assembly and annotation

First, the bottle gourd mitochondrial genome was assembled on the basis of Nanopore long-read data. The long-read sequencing data were directly assembled via Flye software with the default parameters to generate graphical assembly results in GFA format (Kolmogorov et al., 2019). For all assembled FASTA-format contigs, we constructed a library via makeblastdb and subsequently utilized the BLASTn program to identify contig fragments containing mitochondrial genomes via conserved plant mitochondrial genes in *A. thaliana* as query sequences (-evalue 1e-5 -outfmt 6-max_hsps 10-word_size 7-task blastn-short) (Wick et al., 2015). The GFA files were visualized via Bandage software (version 0.8.1), and the mitochondrial contigs were screened against the BLASTn results to generate a sketch of the bottle gourd mitochondrial genome. The long-read and short-read data were compared to the mitochondrial contigs via BWA software (version 0.7.17) and exported by filtering the mitochondrial reads in the alignment (Wick et al., 2017). A Unicycler with the default parameters was used for hybrid assembly, and the bottle gourd mitochondrial genome was ultimately assembled and then visualized via Bandage software (version 0.8.1) (Tillich et al., 2017).

The PCGs of the mitochondrial genome were selected as reference genes for *A. thaliana* (NC_037304) and watermelon (NC_014043.1), and the sequence of the mitochondrial genome was annotated via GeSeq software (version 2.03). The tRNAs of the mitochondrial genome were annotated via tRNAscan-SE software (version 2.0.11). The rRNAs of the mitochondrial genome were annotated via BLASTN software (version 2.13.0) (Chen et al., 2015). Each mitochondrial genome annotation error was manually corrected via Apollo software (version 1.11.8) (Lewis et al., 2002).

### Identification and validation of repeated-mediated recombination events

MISA (version 2.1), TRF (version 4.09) and the REPuter web server (https://bibiserv.cebitec.uni-bielefeld.de/reputer/) were used to identify repeats, including microsatellite sequence repeats, tandem repeats, and scattered repeats (Kurtz et al., 2001; Beier et al., 2017). The results were visualized with Excel (version 2021) and the Circos package (version 0.69-9) (Zhang et al., 2013). On the basis of the bottle gourd mitochondrial genomic data, sequences 500 bp upstream and downstream of the repeat sequences were extracted, and primers were designed using PrimerBLAST (**Supplementary Table S1**). The PCR mixture consisted of 1 μl of DNA, 2 µl of 10 μM forward and reverse primers, respectively, 25 μl of 2× Phanta Max Master Mix, and 20 μl of ddH_2_O. PCR was performed under the following conditions: 94°C for 3 min; 37 cycles of 94°C for 15 s, 55°C for 15 s and 70°C for 15 s; and 72°C for 5 min. Further sequencing of the PCR products of the expected size was performed via the Sanger method. The PCR products of the correct size in the electrophoresis gel were subsequently sent to Sangon Bioengineering Co., Ltd. (Shanghai) for sequencing. SeqMan was used to visualize the sequencing results.

### Sequence transfer and codon preference analysis

The chloroplast genome was assembled via GetOrganelle software (version 1.7.7.0), the chloroplast genome was annotated via CPGAVAS2 software, and the chloroplast genome annotation results were corrected via CPGView software (Shi et al., 2019; Jin et al., 2020). The results were visualized via the Circos package (version 0.69-9) by analyzing the homologous fragments via BLASTN software (version 2.13.0) (Zhang et al., 2013). The protein-coding sequences of the genome were extracted via PhyloSuite software (version 1.1.16) (Zhang et al., 2020). Codon preference analysis of the mitochondrial genome PCGs was performed with Mega software (version 7.0), and relative synonymous codon usage (RSCU) values were calculated (Kumar et al., 2016).

### Phylogenetic analysis

The complete mitochondrial genomes of 36 species from four angiosperm orders (Cucurbitales, Rosales, Fagales, and Fabales) related to bottle gourd were downloaded from the NCBI database (see **Supplementary Table S2** for details on the new information). Common genes were then extracted using PhyloSuite (version 1.2.2) software, multiplex sequence alignment analysis was performed with MAFFT software (version 7.505), and IQ-TREE software (version 1.6.12) was used for phylogenetic analysis. ITOL software (version 6) was subsequently used to visualize the results from the phylogenetic analysis (Katoh and Standley, 2013; Nguyen et al., 2015). The corresponding nucleotide sequences were aligned and concatenated via Maft (version 7.450) and Phylosuite, respectively. The Ka/Ks value for each gene was calculated via the KaKs_calculator. In the results, the Ka/Ks “NA”, which appears when Ks = 0 (in cases with no substitutions in the alignment or 100% match), was replaced with 0.

### Collinearity analysis

On the basis of BLASTN (version 2.10.1, parameter: -word_size 7, E value 1e-5), pairwise comparisons of the cucumber species and six proximal taxa (*Cucurbita pepo, Cucurbita maxima, Lagenaria siceraria, Citrullus lanatus, Luffa acutangula* and *Herpetospermum*) were conducted for *Herpetospermum pedunculosum* and *Momordica charantia*, and homologous sequences greater than 500 bp in length were retained as conserved collinear blocks and plotted via a multiple synteny plot.

### RNA editing events and collinearity analysis

Total RNA was extracted using an RNA Plant Extraction Kit (Chinese Tiangen). The RNA integrity and concentration were measured with a NanoDrop 2000 spectrophotometer (Thermo Scientific, USA) and an Agilent 2100 bioanalyzer (Agilent Technologies, USA), respectively. Sequencing libraries were generated after the removal of rRNA (NEB E7420) via the NEBNext Illumina Superdirected RNA Library Preparation Kit. The 5’ end of each library was phosphorylated and cyclized, and circular amplification was then performed to generate DNA nanospheres. These DNA nanospheres were subsequently loaded onto the DNBSEQ-T7 sequencing system for long noncoding RNA (lncRNA) sequencing. On the basis of the lncRNA sequencing data, transcripts from the mitochondrial genomes were filtered and mapped onto mitochondrial DNA sequences, and differences in DNA and RNA sequences were further assessed via BEDTools software (version 2.30.0) to identify the most read-supported RNA editing events (Quinlan and Hall, 2010). On the basis of the BLAST procedure, comparisons of individual mitochondrial genomes were performed, and homologous sequences longer than 500 bp were preserved as conserved collinear blocks to generate multiple synteny plots. Multiple synteny plots of bottle gourds and related species were created via the MCScanX source program (Wang et al., 2012).

## Results

### Assembly and annotation of the *Lagenaria siceraria* mitochondrial genome

**Figure 1:**
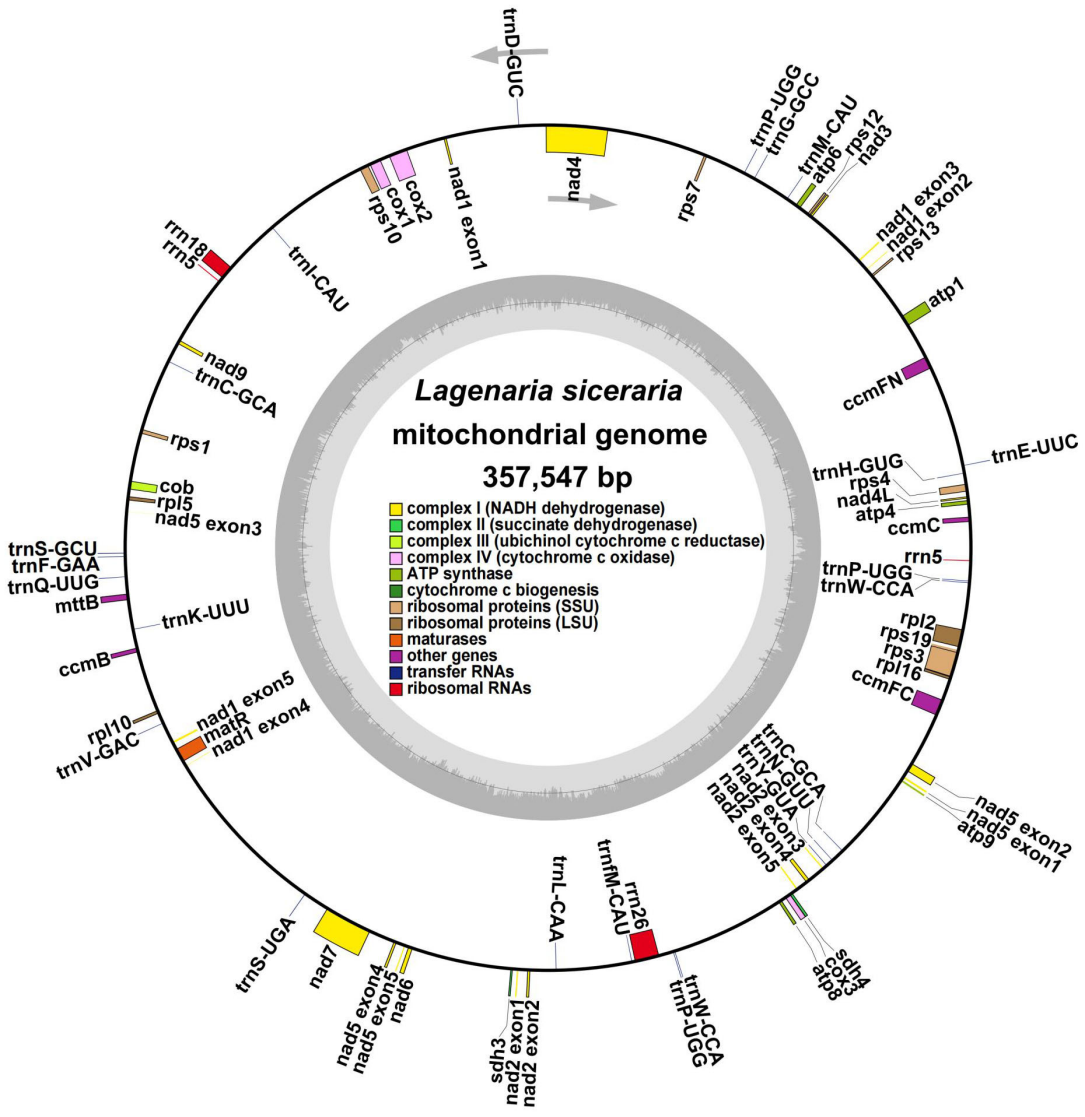
Circular map of the whole *Lagenaria siceraria* mitochondrial genome. Each square represents a different mitochondrial gene, and the same gene is represented by the same color. The exons are denoted as ‘exons’, such as *nad2* exon 1 and *nad2* exon 2, and introns are found between the exons. The arrows indicate the forward and reverse chains.

The bottle gourd mitochondrial genome was assembled using a combination of long- and short-read and long noncoding RNA (lncRNA) sequencing. We generated a total of 7.23 Gb of raw data from three generations of Nanopore platform sequencers. After filtering out connectors, short fragments and low-quality data, a total of 7.14 GB of clean data with an N50 of 20153 bp was generated, and the average length of the reads in the clean data was 12157 bp (**Supplementary Table S3**). A total of 6.63 Gb of raw data were generated via a second-generation Illumina platform sequencer, and after filtering out connectors, short fragments and low-quality reads, a total of 6.61 Gb of clean data with a Q20 of 95.89%, a Q30 of 89.58%, and a GC content of 34.96% were generated (**Supplementary Table S3**). A total of 15.25 Gb of raw data were generated by the lncRNA sequencer, and after the exclusion of connectors, short fragments and low-quality reads, a total of 13.54 Gb of clean data with a Q20 of 96.92%, a Q30 of 92.41%, and a GC content of 41.42% were generated (**Supplementary Table S3**). The second-generation Illumina sequencing data were assembled *de novo* into a unit map, and the continuous-repeat-continuous region was decomposed by third-generation Nanopore long-read sequencing. We then identified the bottle gourd mitochondrial genome as a single circular molecule of 357547 bp with a GC content of 45.03% (**Figure 1**).

Annotation of the bottle gourd mitochondrial genome (**Figure 1**, **Table 1**) revealed 38 unique protein-coding genes (PCGs), including 24 unique mitochondrial core genes, 14 noncore genes, 3 ribosomal RNA (rRNA) genes and 19 transfer RNA (tRNA) genes. The core bottle gourd genes included 5 ATP synthase genes, 9 NADH dehydrogenase genes, 1 cytochrome b gene, 4 cytochrome c biogenesis genes, 3 cytochrome c oxidase genes, 1 maturase gene and 1 protein transport subunit. Fourteen noncore genes, namely, 4 ribosomal protein large subunit genes, 8 ribosomal protein small subunit genes, and 2 succinate dehydrogenase genes, were identified. Among the three rRNA genes, rrn5 was a double-copy gene, and rrn18 and rrn26 were single-copy genes. Among the 19 tRNA genes, trnC-GCA and trnW-CCA were double-copy genes, trnP-UGG was a three-copy gene, and the remaining genes were single-copy genes. We submitted the annotated complete bottle gourd mitochondrial genome to GenBank under accession number OR680814.

**Table 1:** Gene contents of the mitogenome of *Lagenaria siceraria*.

	Gene groups	Names of genes
Core genes	ATP synthase	atp1, atp4, atp6, atp8, atp9
	NADH dehydrogenase	nad1, nad2, nad3, nad4, nad4L, nad5, nad6, nad7, nad9
	Cytochrome b	cob
	Cytochrome c biogenesis	ccmB, ccmC, ccmFC, ccmFN
	Cytochrome c oxidase	cox1, cox2, cox3
	Maturase	matR
	Protein transport subunit	mttB
Noncore genes	Ribosomal protein large subunit	rpl2, rpl5, rpl10, rpl16
	Ribosomal protein small subunit	rps1, rps3, rps4, rps7, rps10, rps12, rps13, rps19
	Succinate dehydrogenase	sdh3, sdh4
rRNA genes	Ribosome RNA	rrn5(×2), rrn18, rrn26
tRNA genes	Transfer RNA	trnC-GCA(×2), trnD-GUC, trnE-UUC, trnF-GAA, trnfM-CAU, trnG-GCC, trnH-GUG, trnI-CAU, trnK-UUU, trnL-CAA, trnM-CAU, trnN-GUU, trnP-UGG(×3), trnQ-UUG, trnS-GCU, trnS-UGA, trnV-GAC, trnW-CCA(×2), trnY-GUA

### Structure and codon preferences of the mitochondrial genome of bottle gourd

**Figure 2:**
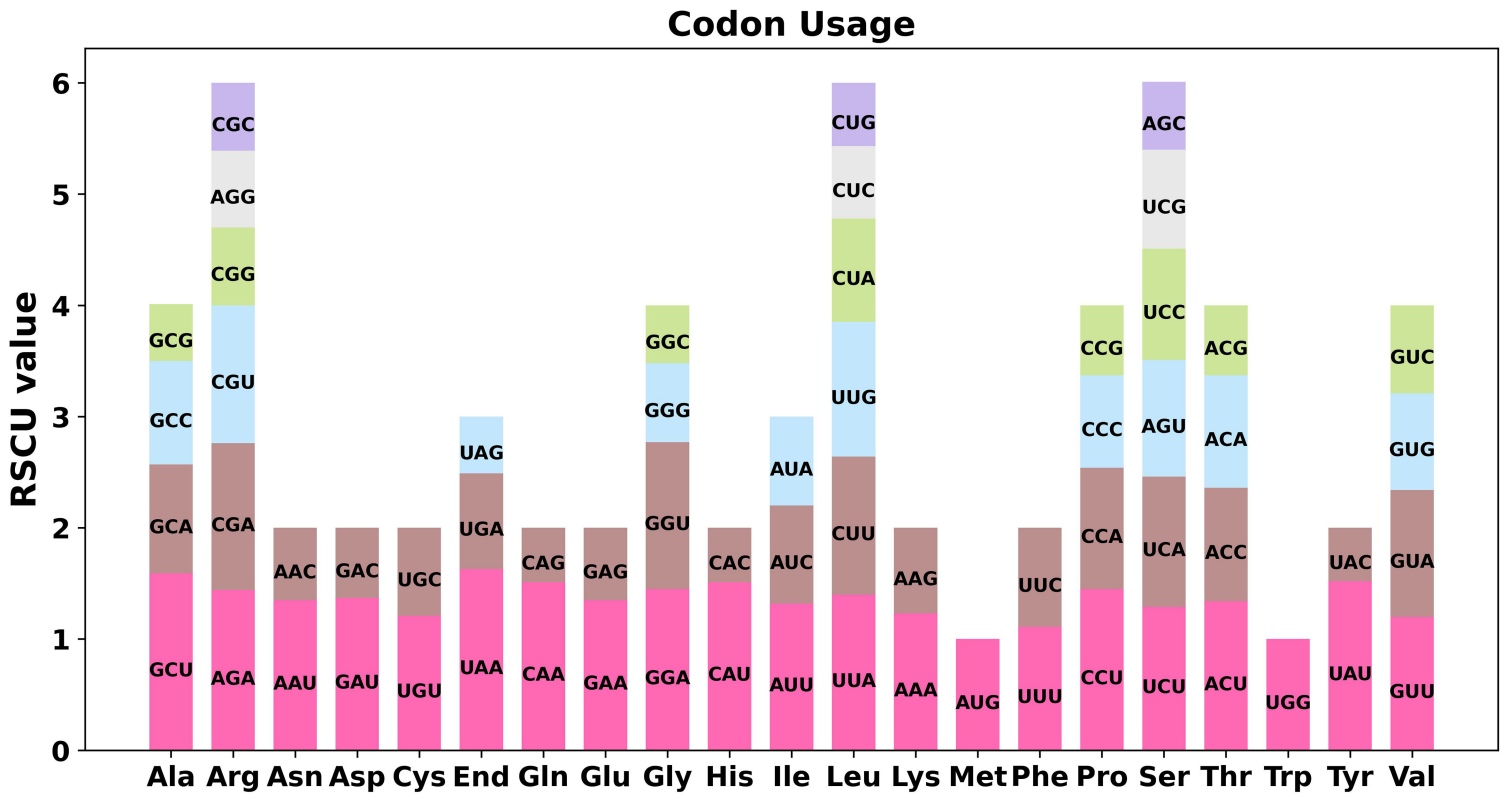
Codon preference analysis of the bottle gourd mitochondrial genome. Different colors represent the RSCU values for different codons for the same amino acid. The abscissa represents the different amino acids, and the ordinate represents the RSCU values for the different codons for each amino acid.

For the 38 PCGs in bottle gourd mitochondria, we conducted a codon preference analysis. **Supplementary Table S4** displays the data on codon utilization by certain amino acids. Codons with RSCU values greater than 1 were considered preferable for certain amino acids. In addition to the starting codons AUG and tryptophan (UGG), which both had RSCU values of 1, the PCGs in bottle gourd mitochondria presented other common preferences for codon use (**Figure 2**). For example, the stop codon showed a greater preference for UAA, with the highest RSCU occurring at 1.63, followed by alanine (Ala), with a preference for GCU with an RSCU value of 1.59. Notably, phenylalanine (Phe) has an RSCU of 1.11 for UUU and thus does not show a strong codon preference.

### Mitochondrial genome repeat analysis

**Figure 3:**
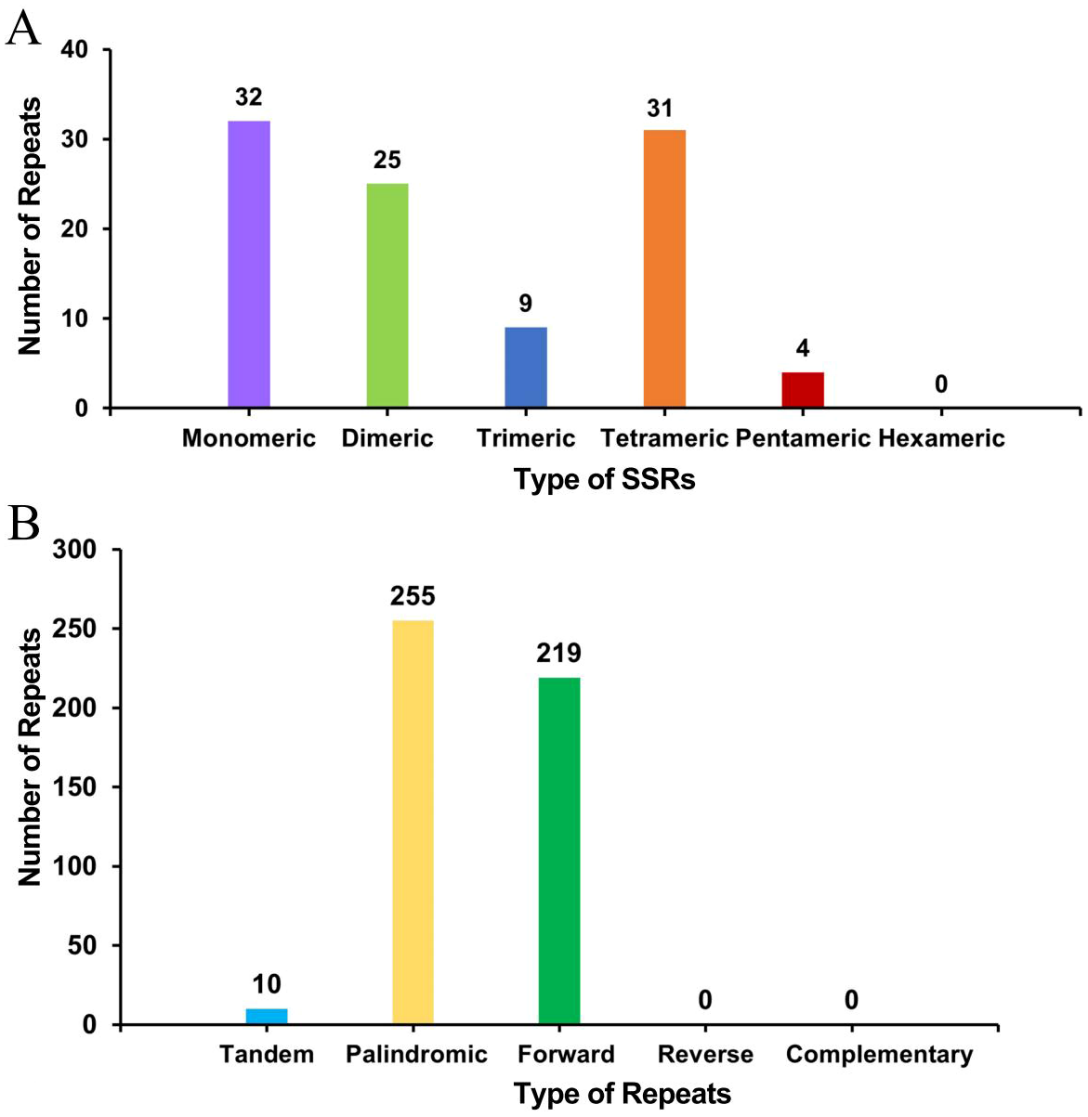
Histogram of the results from the repeat sequence analysis. **(A)** The abscissa represents the type of SSR, and the ordinate represents the number of repeating fragments. **(B)** The abscissa represents the type of repeat sequence, and the ordinate represents the number of repeated fragments.

A total of 101 simple sequence repeats (SSRs) were found in the bottle gourd mitochondrial genome, and monomeric and dimeric SSRs accounted for 56.44% of the total SSRs (**Figure 3A**). Adenine monomeric repeats accounted for 46.88% (15) of the 32 monomeric SSRs. No hexameric SSRs were detected in this mitochondrial genome. A total of 10 tandem repeats with a matching degree greater than 80% and a length between 12 and 39 bp were detected in the bottle gourd mitochondrial genome. Moreover, the analysis revealed 474 pairs of repeats with lengths greater than or equal to 30 bases; 255 and 219 of these were palindromic and forward repeats, respectively, and no reverse repeats or complementary repeats were detected (**Figure 3B**). The longest palindromic repeats had a length of 2349 bp, and the longest forward repeats had a length of 1685 bp.

### Recombination mediated by repeat sequences

**Figure 4:**
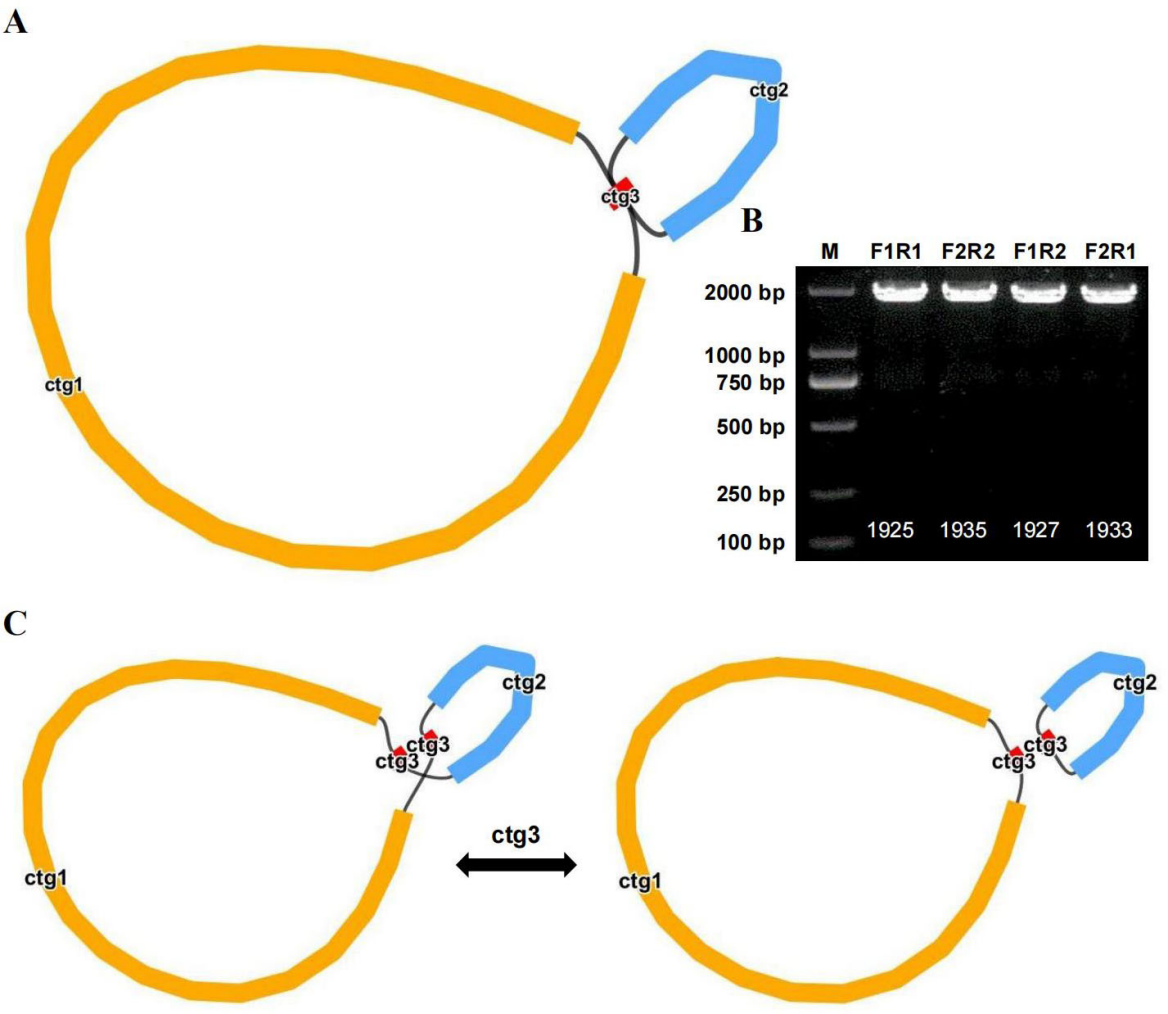
Potential isomers of the bottle gourd mitogenome inferred from PCR experiments and long reads. **(A)** Bottle gourd mitochondrial genome conformation. **(B)** Bottle gourd mitochondrial genome repeat verification. Gel electrophoresis results of PCR products amplified with primers for F1, F2, R1, and R2 using the ctg3 sequence described in **(A)**. The sequences shown in the assembled genome were used to generate PCR products using primers for F1/R1 and F2/R2. The presence of recombinant sequences resulted in the generation of PCR products for F1/F2 and R1/R2 using primers. **(C)** Repeat-mediated (ctg3) conformational recombination of the bottle gourd mitochondrial genome.

We compared long reads to repeat sequences to determine whether the long reads spanned the repeat region and then derived the most likely structure of the mitochondrial genome. The final results revealed that the bottle gourd mitochondrial genome contained 3 nodes (**Figure 4A**). On the basis of the long-read data, one cyclic contig sequence (**Supplementary Figure S1**) was generated after the branching node caused by the repeat sequence (i.e., ctg3) was resolved, and the repeat sequence (node ctg3) was hypothesized to mediate genome recombination and form a structure consisting of two small rings (**Supplementary Figure S1**). To this end, we designed two pairs of PCR primers (F1/R1 and F2/R2) to verify the presence of the identified recombinant products. The ctg3 repeat sequence amplified bands of approximately the expected size before and after the exchange of reverse primers (**Figure 4B**). Sanger sequencing revealed that the PCR products were consistent with the template sequence, suggesting that ctg3-mediated recombination can breakdown the bottle-gourd mitochondrial genome into one large circular molecule and one small circular molecule (**Figure 4C**; **Supplementary Figure S2**). The repeat sequence ctg3 in the bottle gourd mitochondrial genome can mediate chromosomal recombination into two interconverting conformations (GenBank accession number OR680814).

### Intergenomic sequence migration

**Figure 5:**
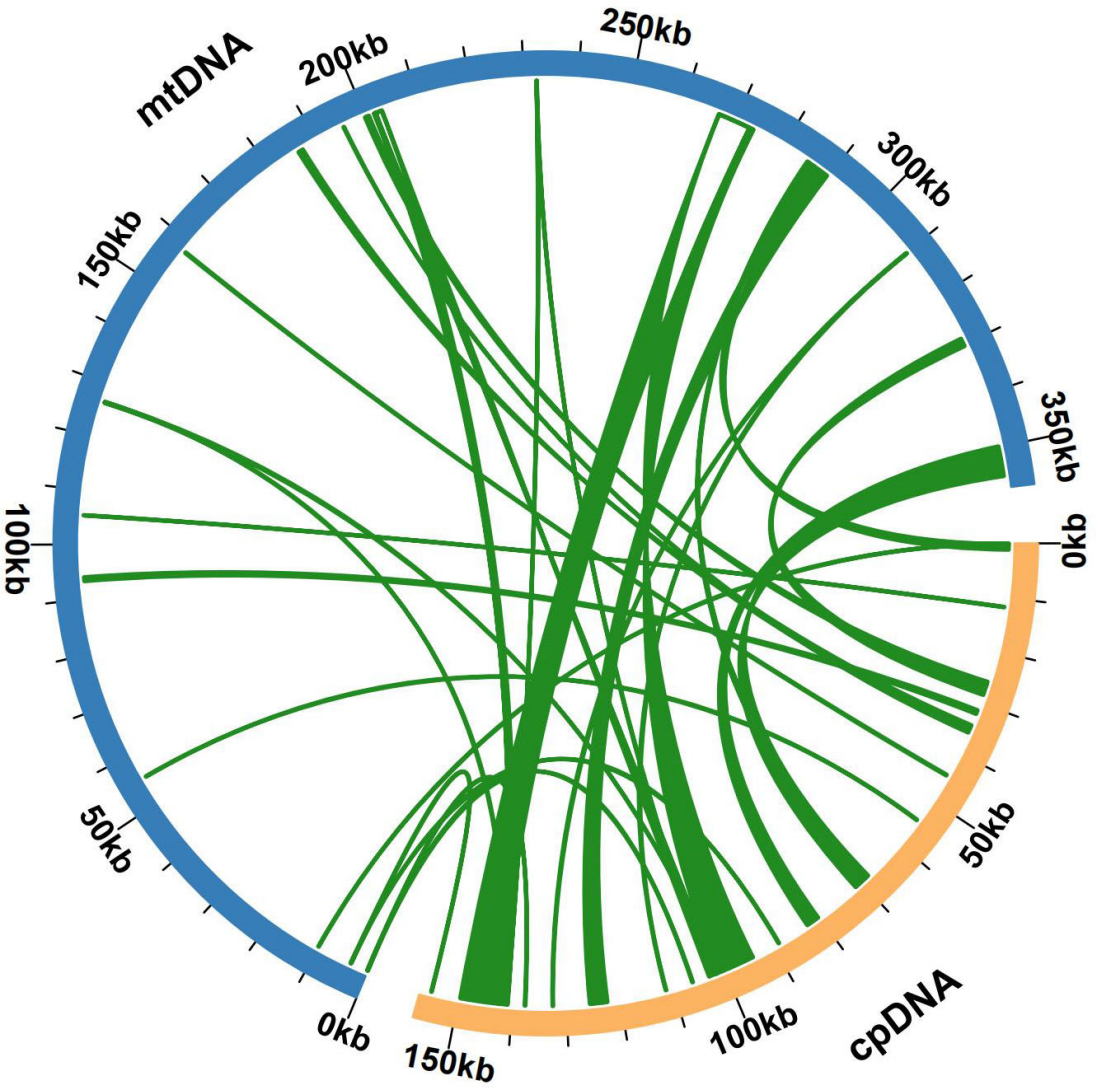
Gene exchange in the chloroplast genome and the bottle gourd mitochondrial genome. The blue arc represents the mitochondrial genome, the yellow arc represents the chloroplast genome, and the genome fragment corresponding to the green connection between the arcs is a homologous fragment. The thickness of the green lines represents the size of the cognate fragments, and the number of lines represents the number of homologous fragments.

Some chloroplast DNA fragments were found to have moved into the mitochondrial DNA during mitochondrial evolution. We also assembled the bottle gourd chloroplast genome, which is composed of a single-loop double-stranded DNA molecule and has a total sequence length of 157121 bp and a GC content of approximately 50.10% (**Supplementary Figure S3**). Sequence similarity analysis revealed that a total of 27 segments, which had a combined length of 23226 bp and represented 6.50% of the length of the mitochondrial genome, were homologous sections of the bottle gourd chloroplast and mitochondrial genomes (**Figure 5**). Mitochondrial plastid DNA (MTPT) 17 was the longest homologous sequence, with a length of 6,184 bp. By annotating the sequences of these homologs, 19 complete genes were found in 27 homologous fragments: 10 PCGs and 9 tRNA genes (**Supplementary Table S5**). The presence of MTPT sequence ligation sites in the mitochondrial genome of bottle gourd was verified, and both end ligation sites of MTPT6, MTPT7, MTPT8, MTPT9, MTPT10, MTPT17 and MTPT18 could amplify bands consistent with the expected size. Sanger sequencing revealed that the PCR product was consistent with the template sequence, confirming that MTPT6, MTPT7, MTPT8, MTPT9, MTPT10, MTPT17 and MTPT18 was present in the mitochondrial genome of bottle gourd (**Supplementary Figure S4**). Small fragments that were partially derived from chloroplasts were also discovered to be subsets of larger sequence fragments or to occur several times in the mitochondrial genome.

### Phylogenetic analysis

**Figure 6:**
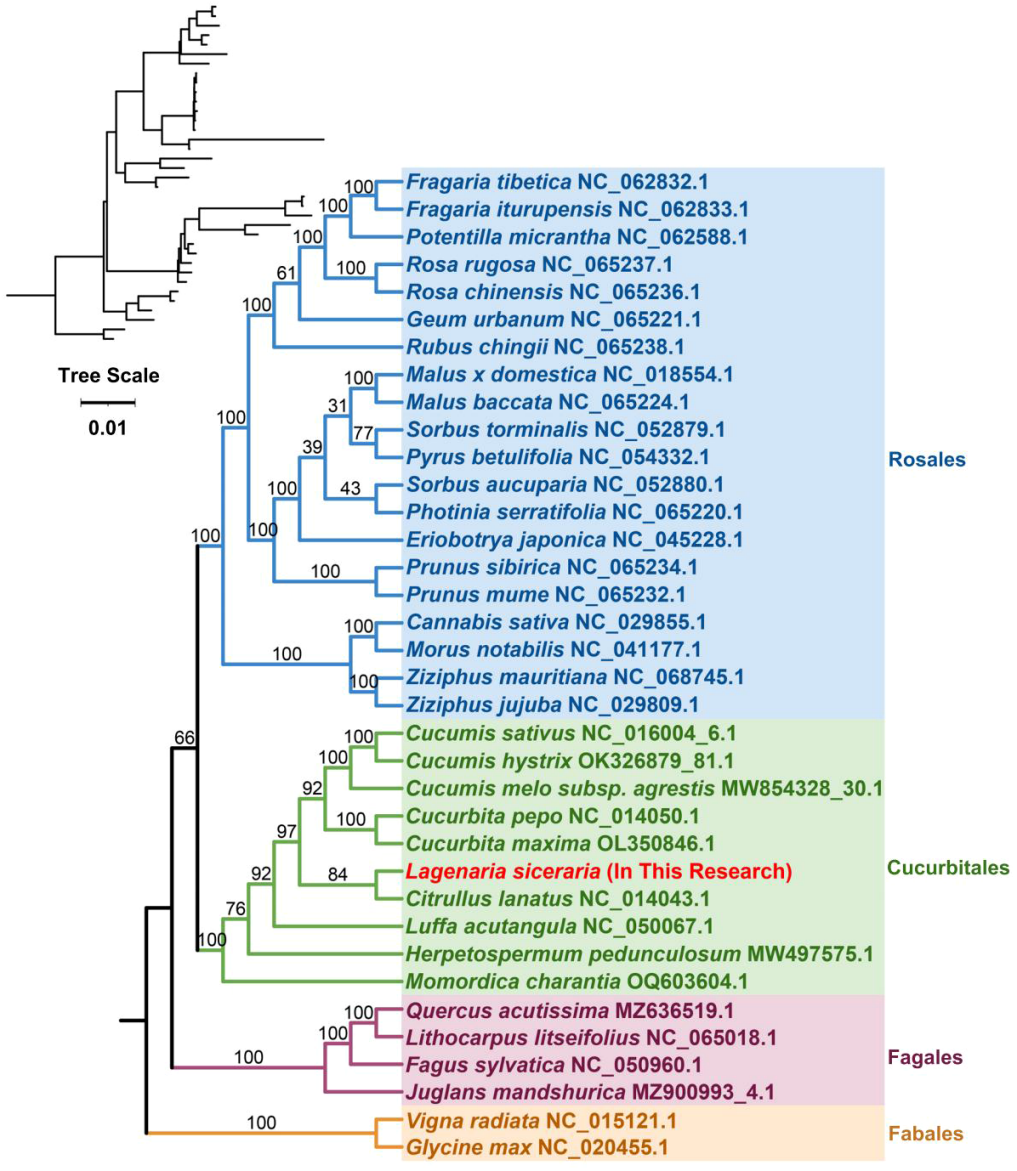
In the phylogenetic analysis of bottle gourd, different colors represent different orders, and red represents the bottle gourd mitochondrial genome. The number on a branch indicates the reliability of the branch, and a higher number indicates increased reliability. The upper left corner shows the evolutionary phylogram diagram; in this diagram, the length of the branch represents the evolutionary distance, and a greater distance indicates a greater change from the state of the ancestor.

The plant mitochondrial genome size and structure are highly variable, but the genes involved are highly conserved. The base replacement rate of PCGs is the most highly conserved among the nuclear, plastid and mitochondrial genomes of plants, but the degree of differentiation is insufficient; therefore, the whole mitochondrial genome is generally not selected as a molecular marker for species phylogenetic analysis and genetic diversity analysis of germplasm resources. However, some protein-coding sequences, such as those of the *COX3* and *CCB203* genes, are often used in such studies. On the basis of the DNA sequences of 26 conserved mitochondrial PCGs, phylogenetic tree reconstruction was performed using 36 species from four orders of angiosperms (Cucurbitales, Rosales, Fagales, and Fabales) (**Supplementary Table S2**). The two mitochondrial genomes of the legume order were used as outgroups. *Cucumis sativus* and *Cucumis hystrix* were located on the most recently diverged branch, and *Cucumis melo* and *Cucurbita pepo* were most recently related to their origin. The bottle gourd belongs to the Cucurbitaceae family of the Cucurbitales order, is grouped on the same branch as *Citrullus lanatus*, and is closely related to *Cucurbita maxima* (**Figure 6**). The results of our study are consistent with the theory that plant mitochondrial genes are highly conserved and that plant mitochondrial genomes are not suitable for phylogenetic studies.

**Figure 7:**
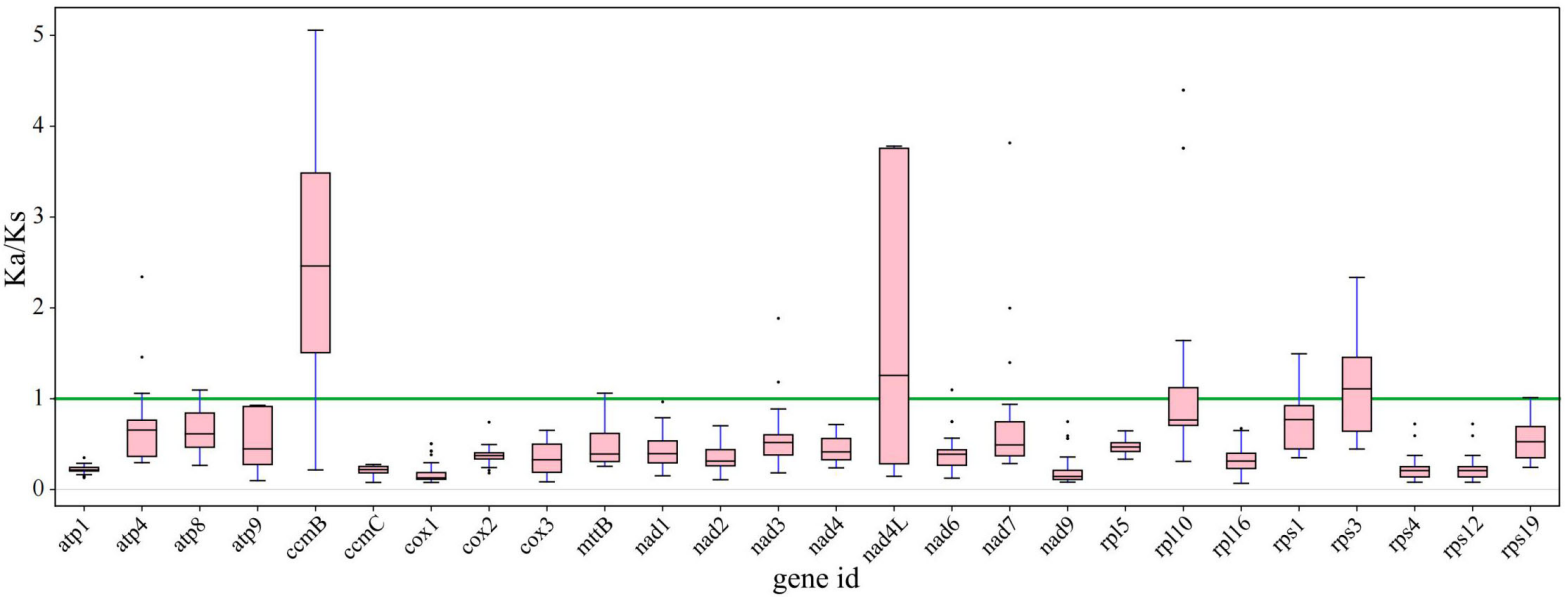
The Ka/Ks ratios of 26 conserved mitochondrial PCGs of 36 species from four orders of angiosperms (Cucurbitales, Rosales, Fagales, and Fabales) were compared with those of bottle gourd.

Synonymous and nonsynonymous nucleotide substitution patterns are very important markers in gene evolution studies. A Ka/Ks ratio (Ka) with synonymous (Ks) substitution rates < 1 indicates purifying selection; Ka/Ks > 1 indicates probable positive selection, whereas Ka/Ks values close to 1 indicate neutral evolution or relaxed selection. We calculated the Ka/Ks ratios of the bottle gourd mitochondrial genome versus those of 35 species from four orders of angiosperms (Cucurbitales, Rosales, Fagales, and Fabales) (**Figure 7**). The Ka/Ks values for most genes were between 0.3~0.9, which means that most of the genes in the bottle gourd mitochondrial genome are under purifying selection. The most conserved genes with average Ka/Ks values between 0 and 0.3, indicating very strong purifying selection pressure, are *atp1, ccmC, cox1, nad9, rps4* and *rps12*. Ectopic overexpression of *Ntatp1* in tobacco can result in cytoplasmic male sterility and seed abortion. Cotton mitochondrial *Ghatp1* can affect ATPase production of ATP and promote epidermal hair and fiber elongation. The *nad3* gene can affect the respiration efficiency and ripening of tomato fruits. However, the Ka/Ks values of most genes suggest that the bottle gourd mitochondrial genome may be affected by purifying selection during the selection and domestication process, and some studies have shown that this selection effect is more obvious in the nuclear genome; therefore, these genomic changes may greatly improve the fruit quality of the bottle gourd. The model averaging method in the KaKs calculator yielded average Ka/Ks values > 1 for *ccmB*, *nad4L* and *rps3*. A Ka/Ks > 1, which is indicative of positive selection, could reflect selection pressures specific to the bottle gourd mitochondrial genome. Alternatively, they could be a sign of increased variability in particular proteins within a broader group of species.

### Collinearity analysis

**Figure 8:**
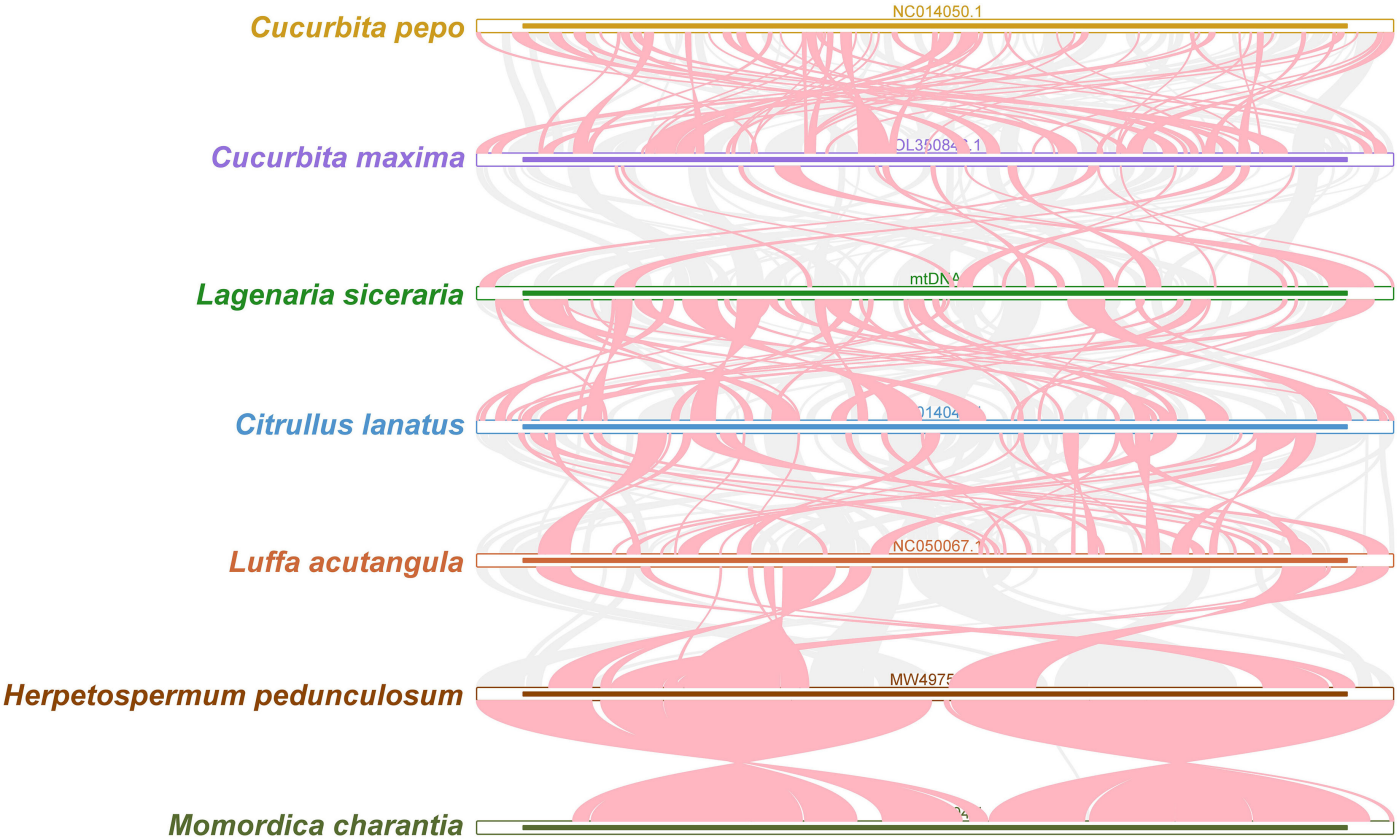
Collinear relationships among the mitochondrial genomes of *C. pepo, C. maxima, L. siceraria, C. lanatus, L. acutangula, H. pedunculosum* and *M. charantia*. The red arcs indicate areas where the inversion occurred, and the gray areas indicate regions with good homology. The different species are represented by different colors, the thickness of the lines represents the size of the homologous fragments, and the number of lines represents the number of homologous fragments.

Red arcs indicate areas where inversion occurred, and gray areas indicate areas with good homology. To better present the results, collinear blocks with lengths of less than 0.5 kb were not retained. Cucurbita species exhibited homologous collinear blocks with 7 closely related species (*C. pepo, C. maxima, L. siceraria, C. lanatus, L. acutangula, H. pedunculosum* and *M. charantia*), but these collinear blocks were shorter. Additionally, sequences that are specific to bottle gourd species and lack similarity with the blank sections of other species were discovered (**Figure 8**). The results revealed that the orders of collinear block arrangement between the mitochondrial genomes of these seven species were inconsistent, and many genome rearrangements were detected between the mitochondrial genomes of bottle gourd species and related species. More collinear gene clusters were found between the bottle gourd mitochondria and the mitochondria of *C. maxima* and *C. lanatus*, which further indicates that bottle gourd is more closely related to *C. lanatus* and *C. maxima* than to the other species (**Figure 8**).

### Bottle gourd mitochondrial RNA editing events

**Figure 9:**
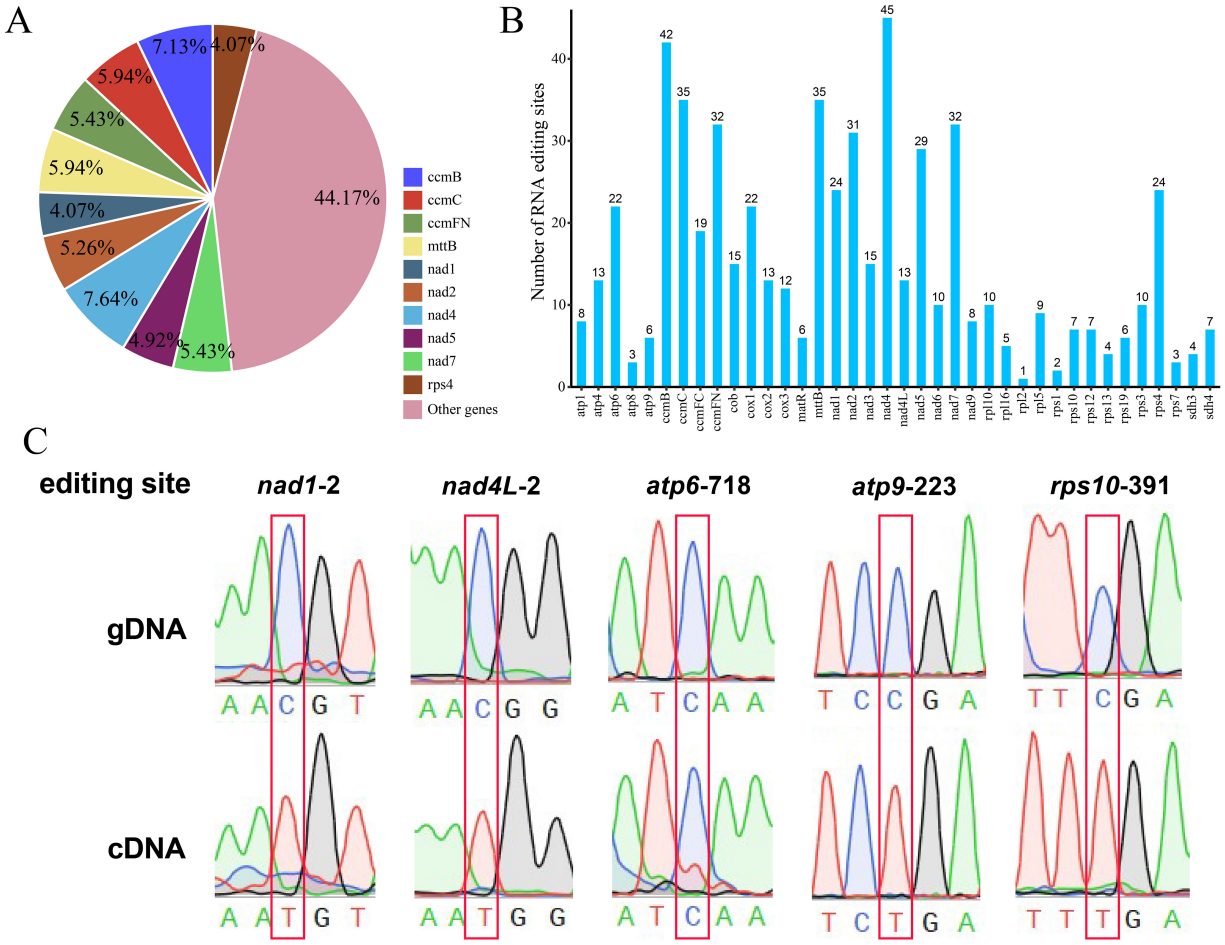
Characteristics of the RNA editing sites identified in the PCGs of bottle gourd. **(A)** Pie chart of RNA editing sites predicted by individual PCGs in the mitochondria of cucurbits. Different RNA editing sites are indicated by different colors, and the percentages are shown in the figure. **(B)** Histogram of RNA editing sites predicted by individual PCGs in the mitochondria of bottle gourd. The horizontal axis represents the different RNA editing sites, and the vertical axis represents the number of each RNA editing site. **(C)** Sequencing peak diagram of mitochondrial RNA editing sites in bottle gourd.

The differences in DNA and RNA sequences were further assessed via BEDTools software (version 2.30.0) to predict mitochondrial genomic RNA editing events in bottle gourd. Thirty-eight distinct PCGs in the bottle gourd mitochondria were used to identify RNA editing events. The 38 mitochondrial PCGs included a total of 589 possible RNA editing events, all of which were C-to-U base edits. Among them, the nad4 gene contained the greatest number (45) of identified RNA editing events, accounting for 7.64% of the total editing events (**Figures 9A, B**). This gene was followed by the ccmB gene, which had 42 RNA editing events, accounting for 7.13% of the total events (**Figures 9A, B**). The *rpl2* gene had only 1 RNA editing event, which was the lowest among all the bottle-gourd mitochondrial genes. The predicted start codon and stop codon editing sites were verified by PCR and Sanger sequencing (**Figure 9C**). The stop codon RNA editing phenomenon was successfully verified at five loci, namely, *nad1*-2*, nad4L*-2*, atp6*-718*, atp9*-223 *and rps10*-391. Among them, the four sites of *nad1*-2, *nad4L*-2, *atp9*-223 and *rps10*-391 have high editing efficiency, and the C base is completely replaced by with T base.

## Discussion

The energy that plants require for growth and development is provided by their mitochondria, which serve as their power plants (Liu et al., 2023). The assembly of plant mitochondrial genomes is incredibly difficult, especially when next-generation sequencing assembly methodologies are utilized, because plant mitochondrial genomes exhibit vast differences in size, sequence order, and repeat content (Attia et al., 2021; Kang et al., 2021; Yang et al., 2021). In recent years, research on the bottle gourd nuclear genome has greatly promoted the study of the evolution and function of bottle gourd genes (Ma et al., 2022). However, bottle gourd mtDNA has not yet been studied; thus, the lack of representative and genetically clear bottle gourd mtDNA represents a major obstacle in the field (Hansen et al., 2007; Cui et al., 2021; Niu et al., 2023; Zhou et al., 2023). Despite notable variances in mitochondrial genome size and structure among various Cucurbitaceae species, previous research has indicated that the mitochondrial genomes of Cucurbitaceae species are circular (Hansen et al., 2007; Cui et al., 2021; Niu et al., 2023; Zhou et al., 2023). For example, the cucumber mitochondrial genome has an especially large main ring and a relatively small subcyclic structure that is approximately 1685 kb in length (Niu et al., 2021). Watermelons and zucchini have single rings of 379 kb and 983 kb, respectively (Cui et al., 2021). The mitochondrial gene of bitter melon is a single loop of 331 kb (Niu et al., 2023). With a size of 2740 kb, the mitochondrial genome of melon is the largest in the Cucurbitaceae family (Chakraborty et al., 2018). However, the cucumber mitochondrial gene has three circular chromosomes that have been mapped and have lengths of 1110 kb, 110 kb, and 92 kb (Kim et al., 2006). Therefore, a thorough study of plant mitochondrial genomes will lead to new information on mitochondrial genome evolution and molecular function. We used Illumina and Oxford Nanopore Technology sequencing data to sequence and assemble the 357-kb circular bottle gourd mitochondrial genome, which is similar in size to the mitochondrial genomes of watermelon and bitter melon (**Figure 1**). This genome provides important information for the molecular breeding of bottle gourds and can also serve as a reference genome for other species in the Cucurbitaceae family.

Consistent with the findings of previous reports, we found that the mitochondrial genome of Cucurbitaceae crops contains complex I~V genes (nad, sdh cob and cox), cytochrome C biosynthesis genes (ccm), ribosomal protein genes (rpl), matR genes, mttB genes, 3 rRNA genes, and 13~24 tRNA genes (bottle gourd contains 19 genes) (Hansen et al., 2007; Cui et al., 2021; Niu et al., 2023; Zhou et al., 2023). The protein-encoding genes of different crop species in the Cucurbitaceae family were relatively conserved, and the number of genes was similar. *rpl10* is absent in dicots such as *Arabidopsis thaliana*, *Brassica napus* and *Beta vulgaris*; thus, its function needs to be exercised by nuclear genes (Kubo and Arimura, 2010). The loss of *rps19* in cucumber and melon during the evolution of Cucurbitaceae may be due to its transfer into the nuclear genome or its function as a pseudogene, but no loss of rpl genes was found in bottle gourd. The proportion of genetic coding sequences and intron sequences in the mitochondrial genome of Cucurbitaceae species, such as bottle gourd, cucumber, melon, and *Cucurbita pepo*, is inversely proportional to the mitochondrial genome size (Hansen et al., 2007; Cui et al., 2021; Niu et al., 2023; Zhou et al., 2023). This may be because the coding sequences and intron sequences in the Cucurbitaceae mitochondrial genome are relatively conserved and the changes in length are small, whereas the changes in length between genes in the mitochondrial genome are large. The differences in tRNA in the mitochondrial genome of Cucurbitaceae crops were significant, and the tRNA genes in the melon mitochondrial genome (21) were the most common among Cucurbitaceae crops, whereas the tRNA genes in *Cucurbita pepo* were the least common (13). *trnH-GUG*, *trnL-CAA* and *trnN-GUU* are tRNA genes unique to melon and bottle gourd, and trnD-GUC, trnM-CAU, and trnW-CCA, three tRNAs, are present only in bottle gourd, cucumber and melon; these genes may have been obtained from the bottle gourd, cucumber and melon nuclear or chloroplast genomes during evolution. *trnK-UUU* is a tRNA gene that is unique to bottle gourd, watermelon and *Cucurbita pepo*, and both melon and cucumber may have lost the *trnK-UUU* gene during evolution.

The intergenic region of the mitochondrial genome is composed mainly of homologous mitochondrial genome sequences, repeat sequences, homologous chloroplast genome sequences, and homologous nuclear genome sequences (Attia et al., 2021; Kang et al., 2021; Yang et al., 2021). Although only 33% of the sequences in the mitochondrial genomes of the melon genus share a homologous relationship with those of other plants, more than 50% of the melon mitochondrial genome sequences are homologous to those of other plants (Cui et al., 2021). The mechanism of sequence migration between organelle genomes and the patterns of gene expression transmitted by migrating fragments in new genes remain mostly unclear, but it is speculated that further improvement of the bottle gourd genome-wide project will yield beneficial innovations for solving these problems. Repeat amplification is frequently thought to be one of the causes of plant mitochondrial genome expansion. The bottle gourd mitochondrial genome contained a total of 101 SSRs and 474 pairs of repeats longer than or equal to 30 bp, and the longest palindromic repeat and the longest forward repeat measured 2349 bp and 1685 bp, respectively (**Figure 3**). Only approximately 23 kb of the bottle gourd mitochondrial genome sequence was homologous to the chloroplast genome sequence, whereas approximately 113 kb of the chloroplast homologous sequence was observed in *C. pepo*, suggesting that chloroplast homologous sequences were not the cause of the expansion of the bottle gourd mitochondrial genome (Ma et al., 2022). Therefore, on the basis of the results from this study, it can be preliminarily speculated that the expansion of the bottle gourd mitochondrial genome was due to increases in homologous sequences, repeat sequences and nuclear genome homologous sequences.

Short-sequence duplication is thought to be an important mediator of chloroplast genome recombination in some algae and lower plants (Ogihara et al., 1988). Although repeat-mediated recombination yields a dizzying array of genomic conformations, in the mitotic genomes of *Salvia* and *Scutellaria skullcap*, repeat sequences can mediate homologous recombination, leading to genomic conformational alterations (Li et al., 2021; Yang J. X. et al., 2023). We resolved repeats (i.e., ctg3) on the basis of long-read data, which may mediate the recombination of the bottle gourd mitochondrial genome and lead to the formation of two small loops. Sanger sequencing revealed that the PCR products were consistent with the template sequence, suggesting that ctg3-mediated recombination can break the bottle-gourd mitochondrial genome into one large circular molecule and one small circular molecule (**Figure 4**). The results demonstrated that the repeat sequence ctg3 in the bottle gourd mitochondrial genome can mediate chromosomal recombination into two interconverting conformations. This finding also suggested that repeat-mediated recombination is the driving force for the structural diversification of plant mitotic genomes.

The inconsistent order of the colinear arrangement suggested that the mitochondrial genome of Cucurbita species underwent extensive rearrangement, which may have contributed to its evolution and diversification (Lee and Park, 2022). After transcription, RNA editing events alter information, particularly at transcription sites, and terrestrial plants frequently undergo mitochondrial RNA editing events (Wu and Chaw, 2022). Additionally, the angiosperm mitochondrial genome has certain conserved RNA editing sites (Grienenberger, 2009). A growing body of evidence suggests that RNA editing by mitochondria is associated with important cultivation traits in plants. For example, RNA editing at the C1292 and C1415 loci of the cotton mitochondrial *Ghatp1* gene can affect ATPase production of ATP and promote epidermal hair and fiber elongation, whereas decreased RNA editing of the *nad3* and *sdh4* genes in tomato can disrupt mitochondrial biological functions, reduce fruit respiration efficiency, and ultimately inhibit tomato fruit ripening (He et al., 2018; Yu et al., 2019). RNA editing is a posttranscriptional regulatory process that occurs mainly in the chloroplasts and mitochondria of plants and is an important means of maintaining the normal biological functions of chloroplasts and mitochondria. The main type of mitochondrial gene RNA editing is cytosine to uracil (C-to-U), which occurs in protein-coding regions, rarely occurs in noncoding regions, and acts on the first and second sites of codons (Alverson et al., 2010). The number of editing sites varies greatly among species; for example, 11 RNA editing sites are found in the mitochondrial genome of *Physcomitrium patens*, while 692 RNA editing sites are found in the mitochondria of cotton. The role of RNA editing is usually to maintain the conservation of amino acid sequences of important functional proteins in organelles, but sometimes, it can also affect the translation of important genes, such as those that encode the production of new start codons or terminators, thereby altering the length and normal function of proteins. The RNA editing times for different genes differ among Cucurbitaceae crops, and ribosomal protein genes (*rpl2, rps1* and *rps7*) undergo less RNA editing than other genes do, whereas the *mttB*, *ccmB* and *ccmFN* genes have greater RNA editing times, which is consistent with our findings (Alverson et al., 2010). For example, most of the nonsynonymous edits in the *ccmFC*, *cob*, *matR* and *mttB* genes were fully edited in *Cucurbita pepo* and partially edited in watermelon and bottle gourd. Therefore, understanding RNA editing in plant mitochondria is crucial for elucidating the function of plant mitochondrial genomes. However, because RNA editing plays a complex and unclear role in plant mitochondrial genomes, additional research is needed to fully understand its regulatory mechanism. The 589 mitochondrial RNA editing sites identified in this study can inform future research related to RNA editing in bottle gourd.

## Conclusions

In this study, the bottle gourd mitochondrial genome was assembled and annotated, revealing a genome size of 357547 bp and 38 PCGs. One pair of repeats was confirmed by PCR amplification and Sanger sequencing to facilitate homologous recombination into one main conformation and one small conformation in the mitochondrial genome. Evolutionary analysis revealed that the bottle gourd mitochondrial genome was conserved, and collinearity analysis revealed that it underwent many rearrangements with the genomes of related species. Moreover, 27 homologous portions of the bottle gourd chloroplast and mitochondrial genomes, which totaled 23226 bp or 6.50% of the length of the mitochondrial genome, were identified. Thirty-eight mitochondrial PCGs were found to include a total of 589 possible RNA editing sites, all of which were altered from base C to U. In conclusion, we assembled and annotated a Cucurbitaceae mitochondrial genome, which advances our understanding of the Cucurbitaceae genome, lays a foundation for elucidating the phylogenetic relationships of Cucurbitaceae and emphasizes the need for further sequencing of the chloroplast, mitochondrial and nuclear genomes of additional Cucurbitaceae and closely related wild species.

## Data Availability

The data presented in the study are deposited in the NCBI repository, accession number PRJNA1026380.

## Supplementary material

Supplementary Figure 1
*Lagenaria siceraria* mitochondrial genome assembly results and recombinant structure.

Supplementary Figure 2 Sanger sequencing results of *Lagenaria siceraria* mitochondrial genome repeats.

Supplementary Figure 3 Circular map of the whole *Lagenaria siceraria* chloroplast genome.

Supplementary Figure 4
*Lagenaria siceraria* particle MTPT verification.
