# Supplementary figures and images for: The de novo assembly and characterization of the complete mitochondrial genome of bottle gourd (Lagenaria siceraria) reveals the presence of homologous conformations produced by repeat-mediated recombination
Source: Front Plant Sci. 2024 Aug 12;15:1416913. doi: 10.3389/fpls.2024.1416913 (PMC11345175; doi:10.3389/fpls.2024.1416913)

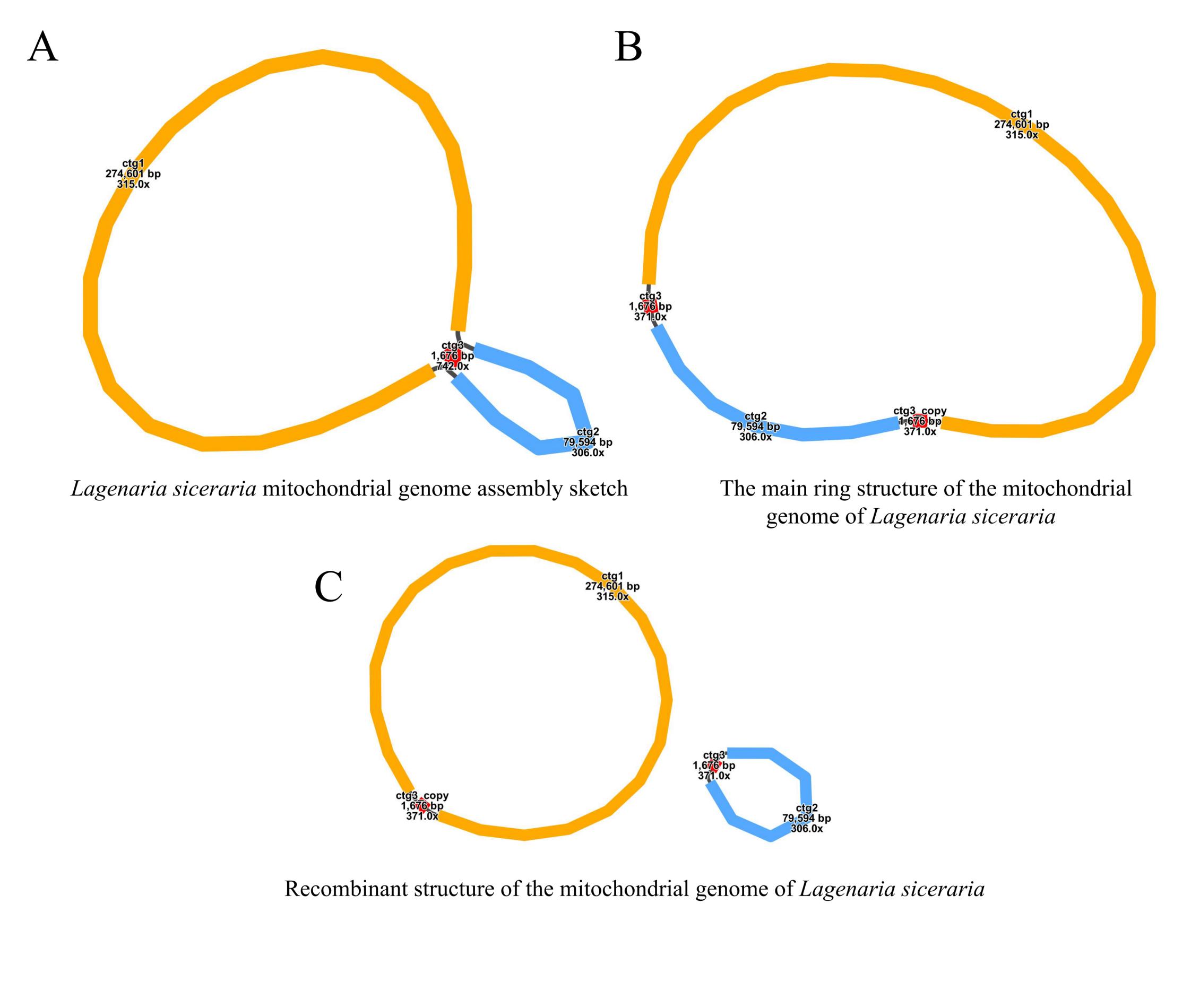

Supplement: Supplementary Figure 1 — Lagenaria siceraria mitochondrial genome assembly results and recombinant structure. [file Image1.jpeg]

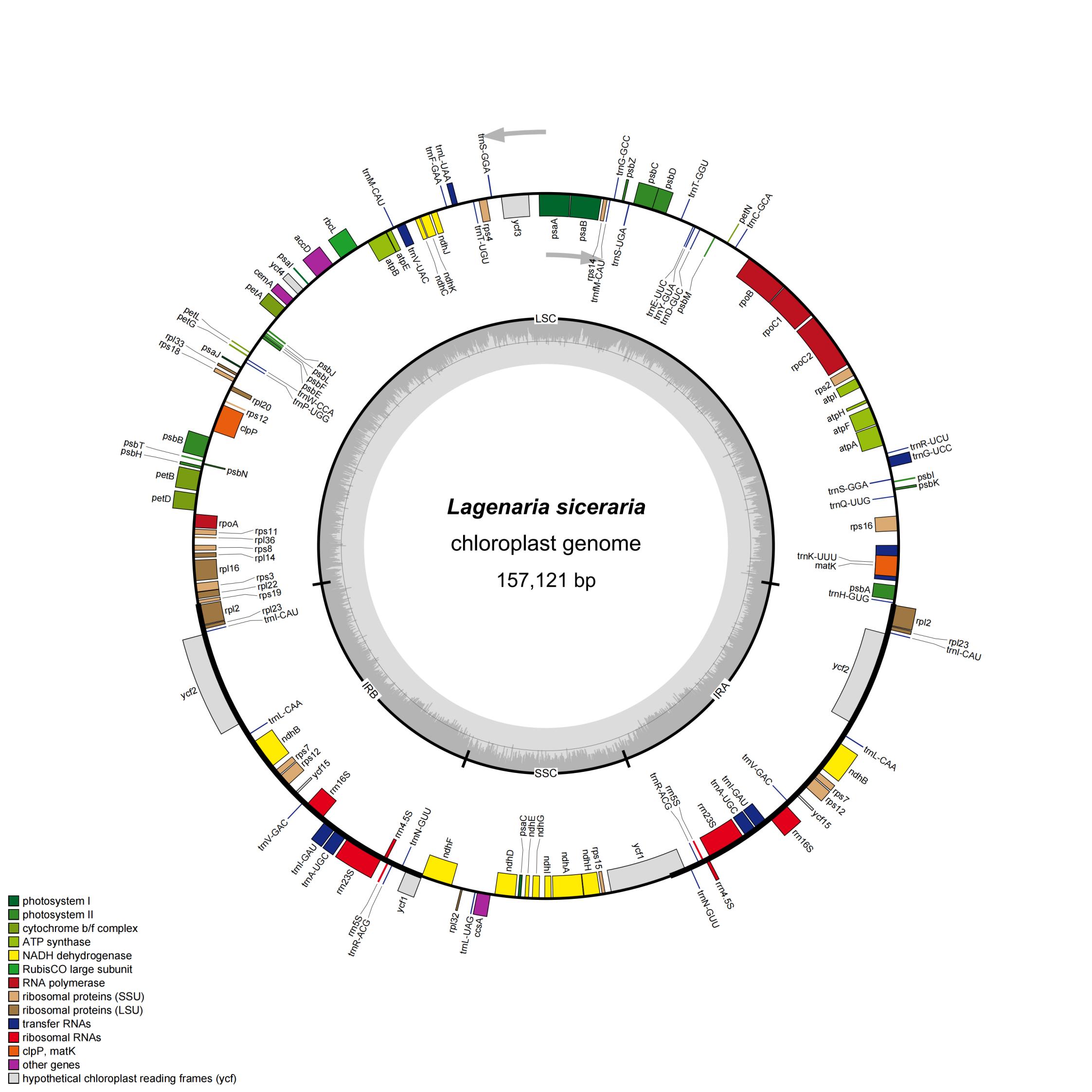

Supplement: Supplementary Figure 2 — Sanger sequencing results of Lagenaria siceraria mitochondrial genome repeats. [file Image2.jpeg]

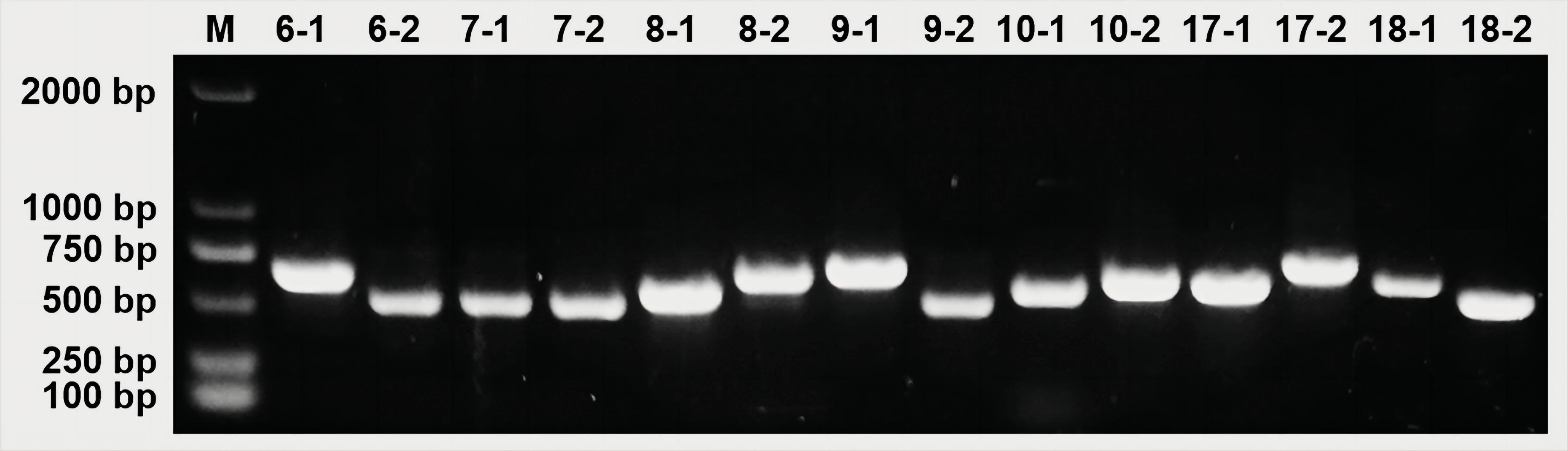

Supplement: Supplementary Figure 3 — Circular map of the whole Lagenaria siceraria chloroplast genome. [file Image3.jpeg]

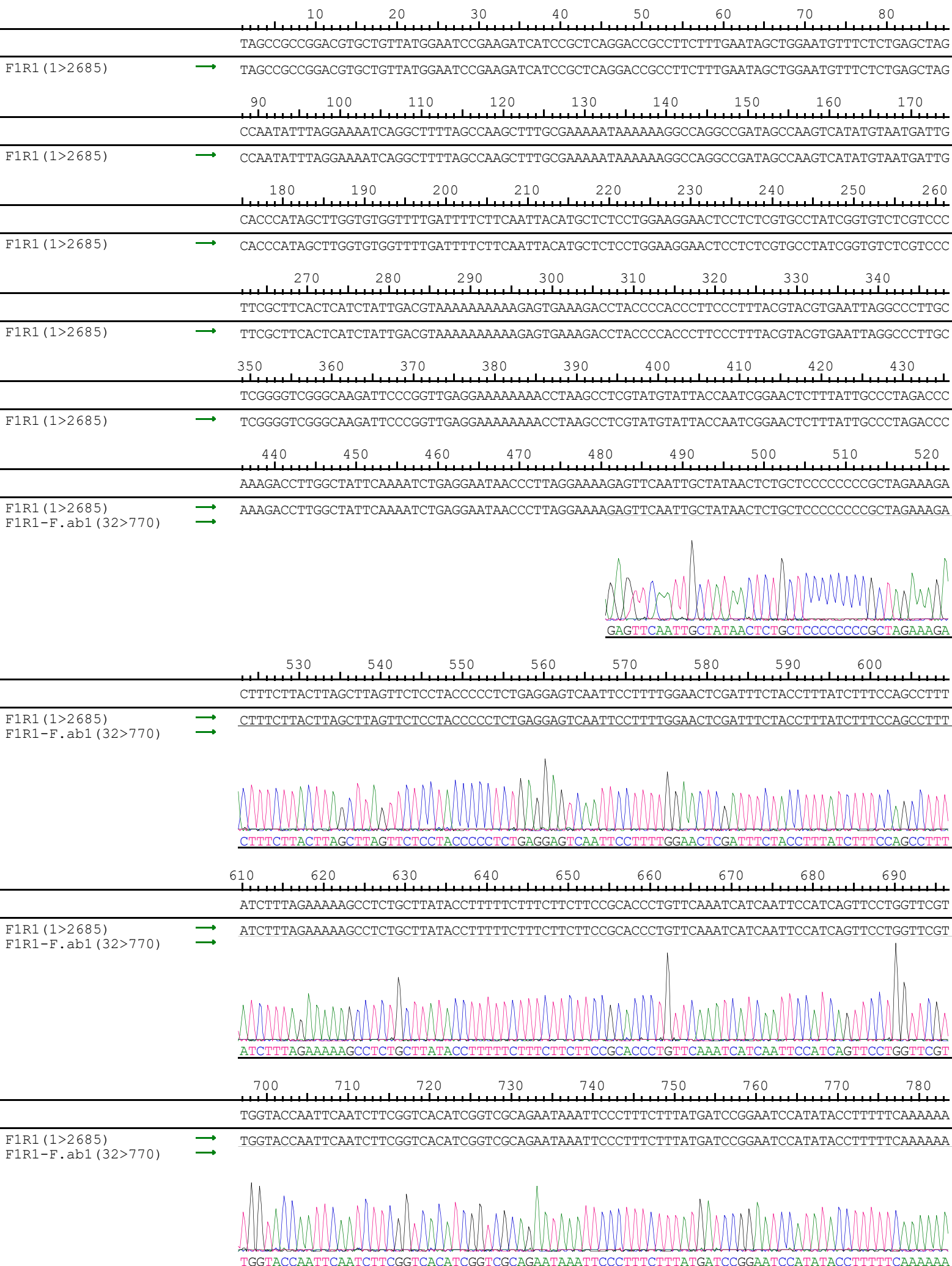

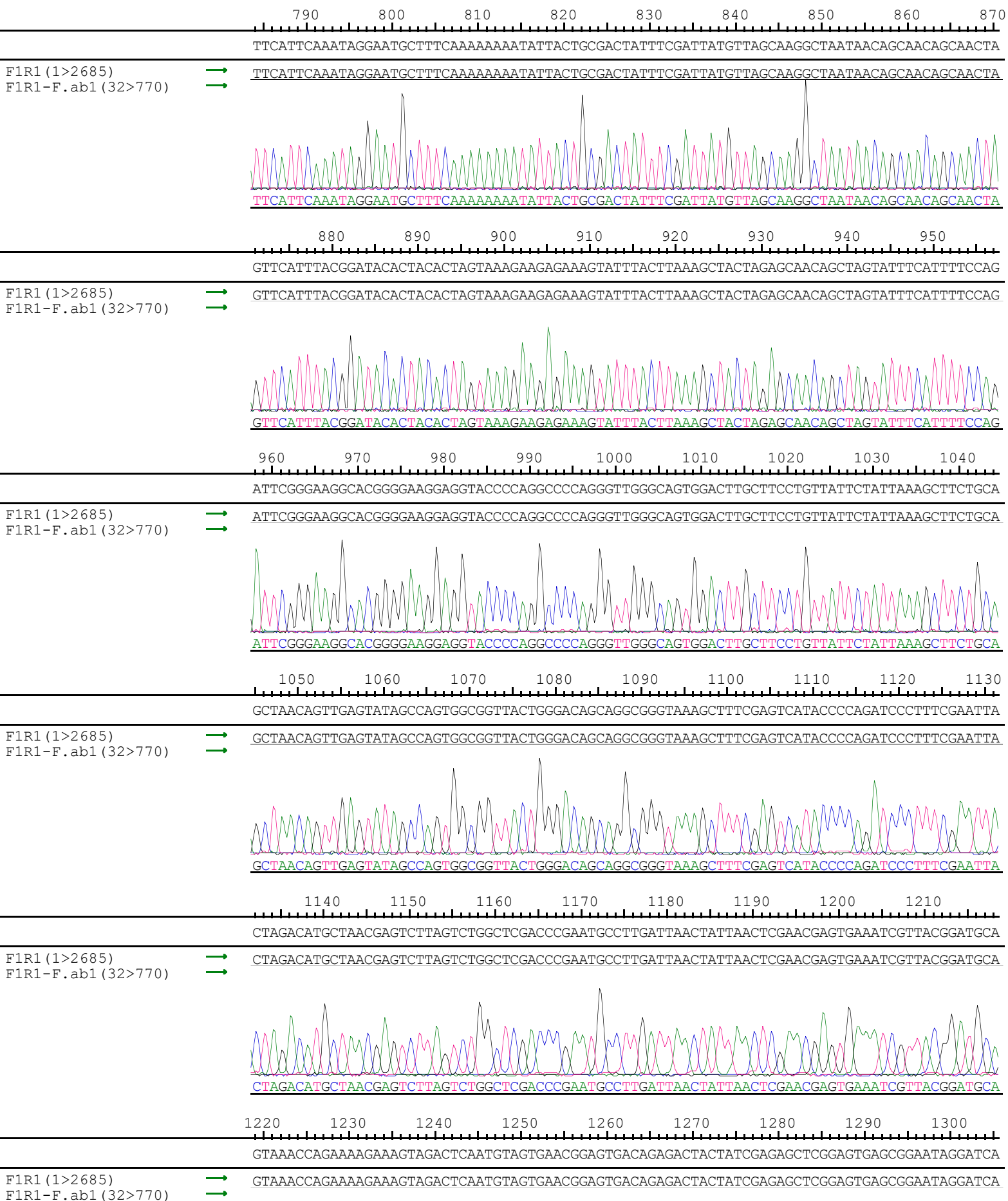

^  
G

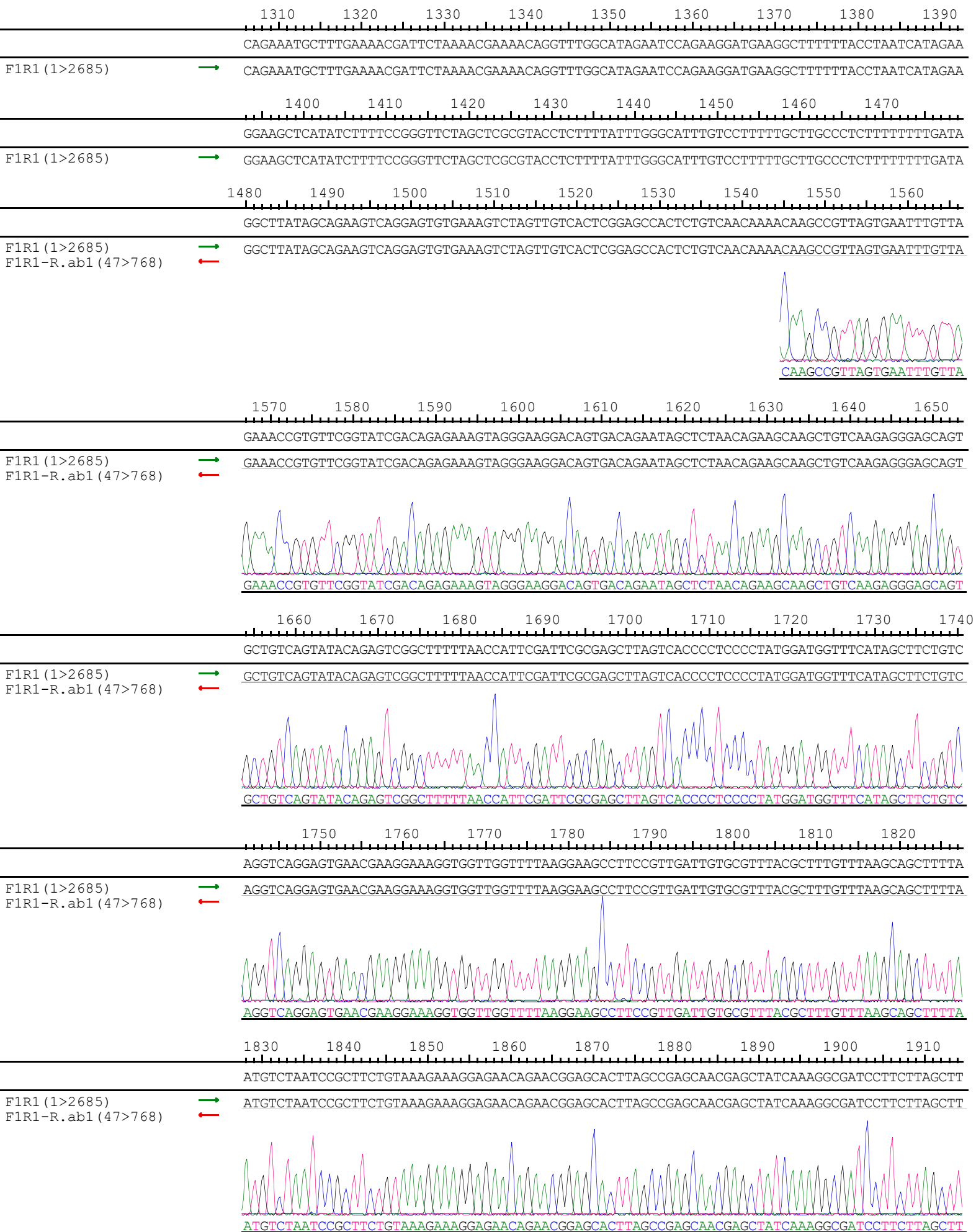

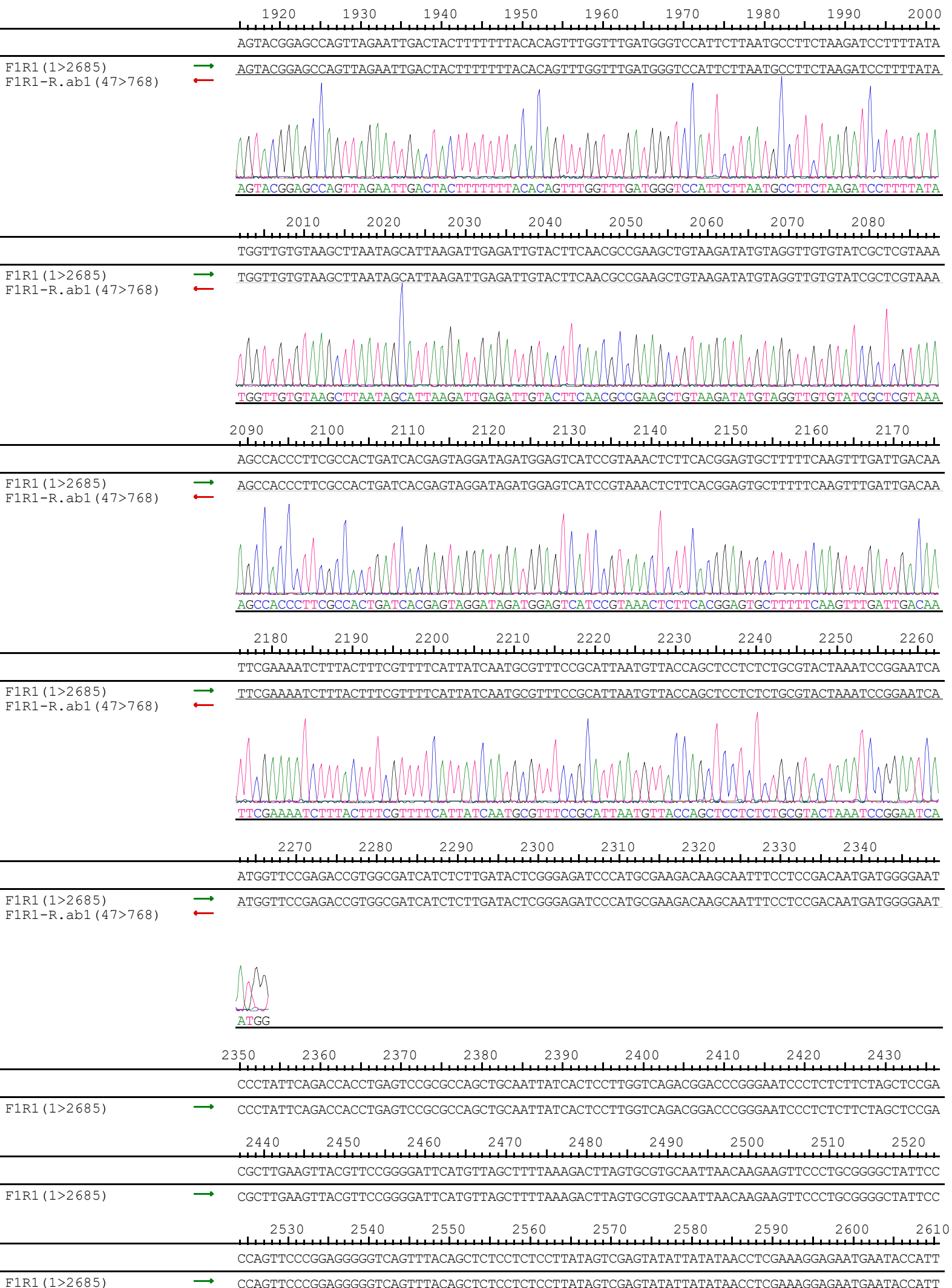

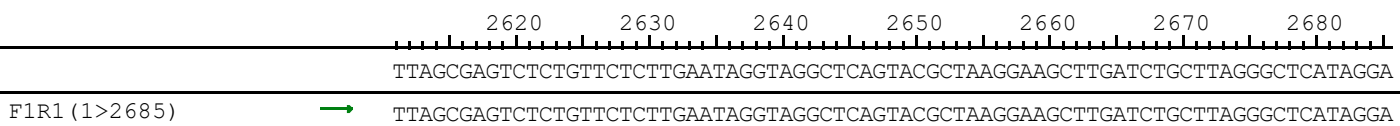

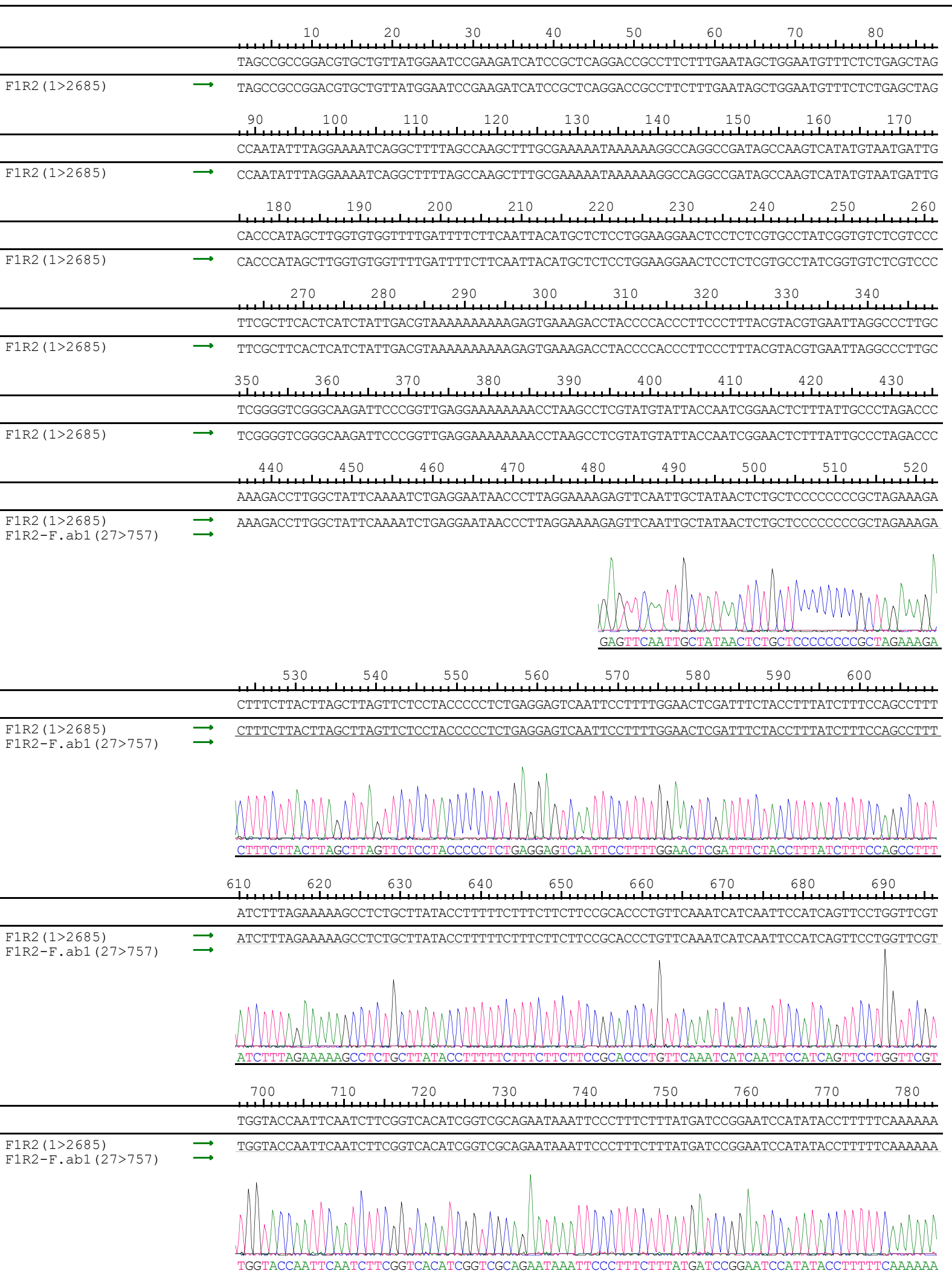

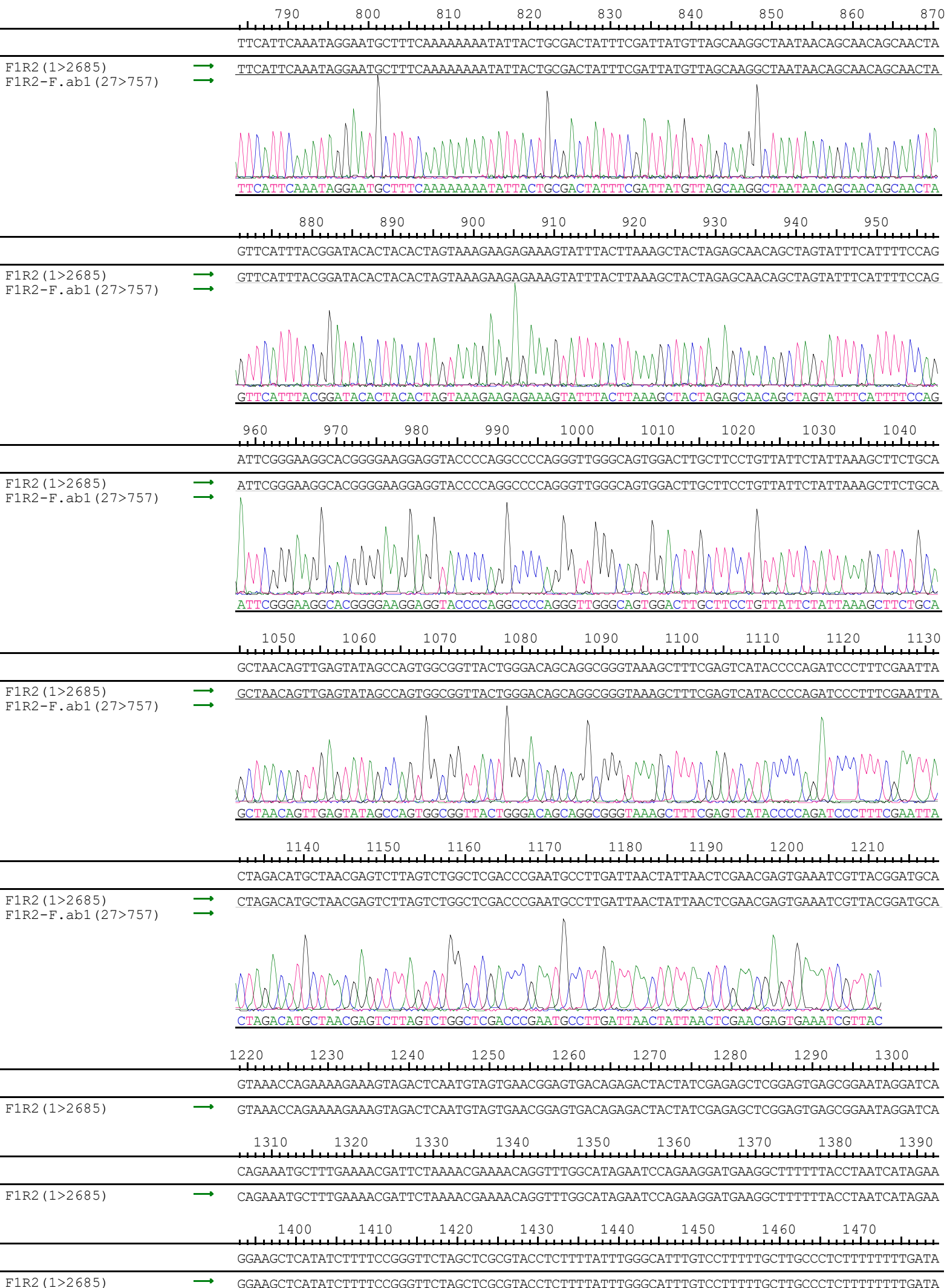

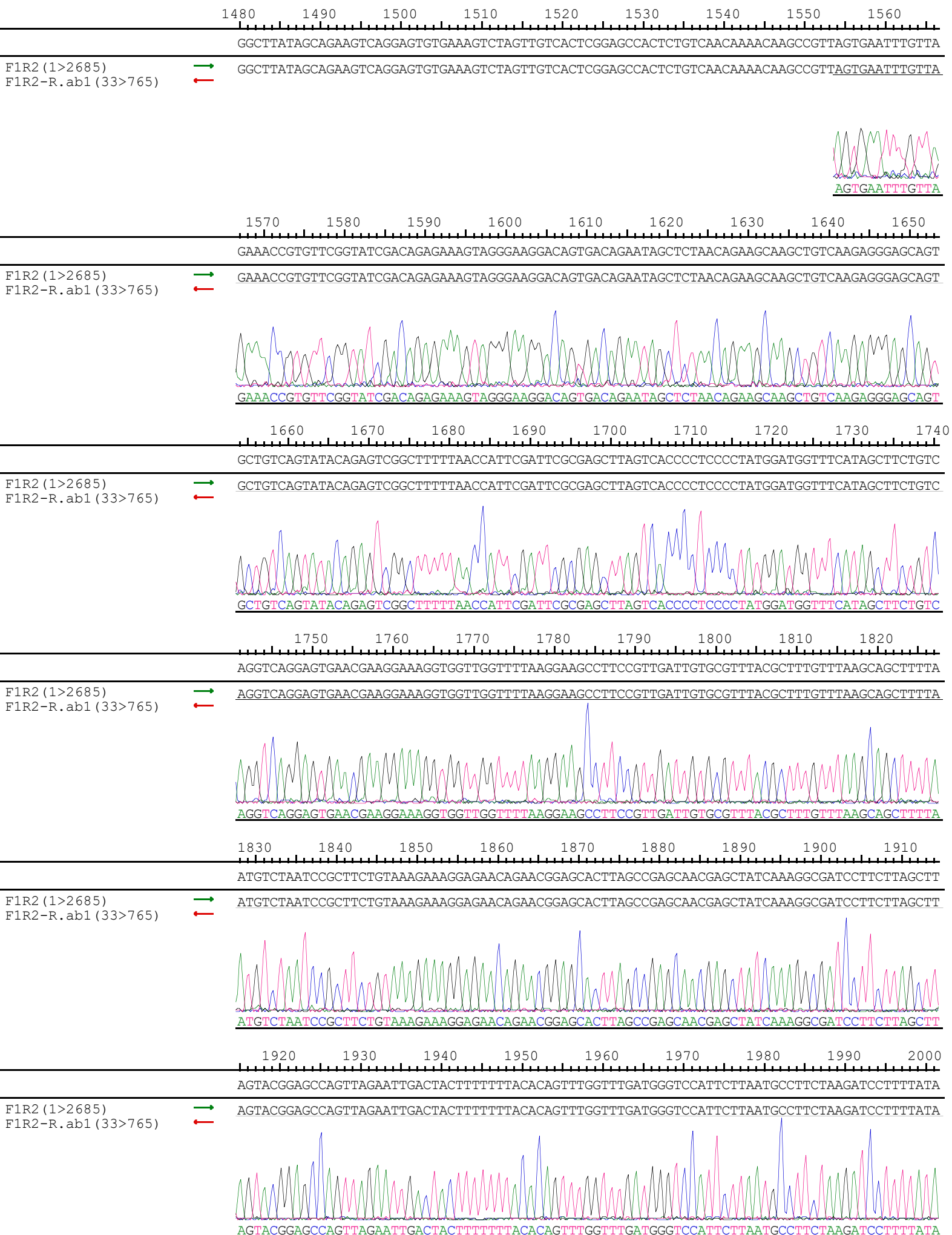

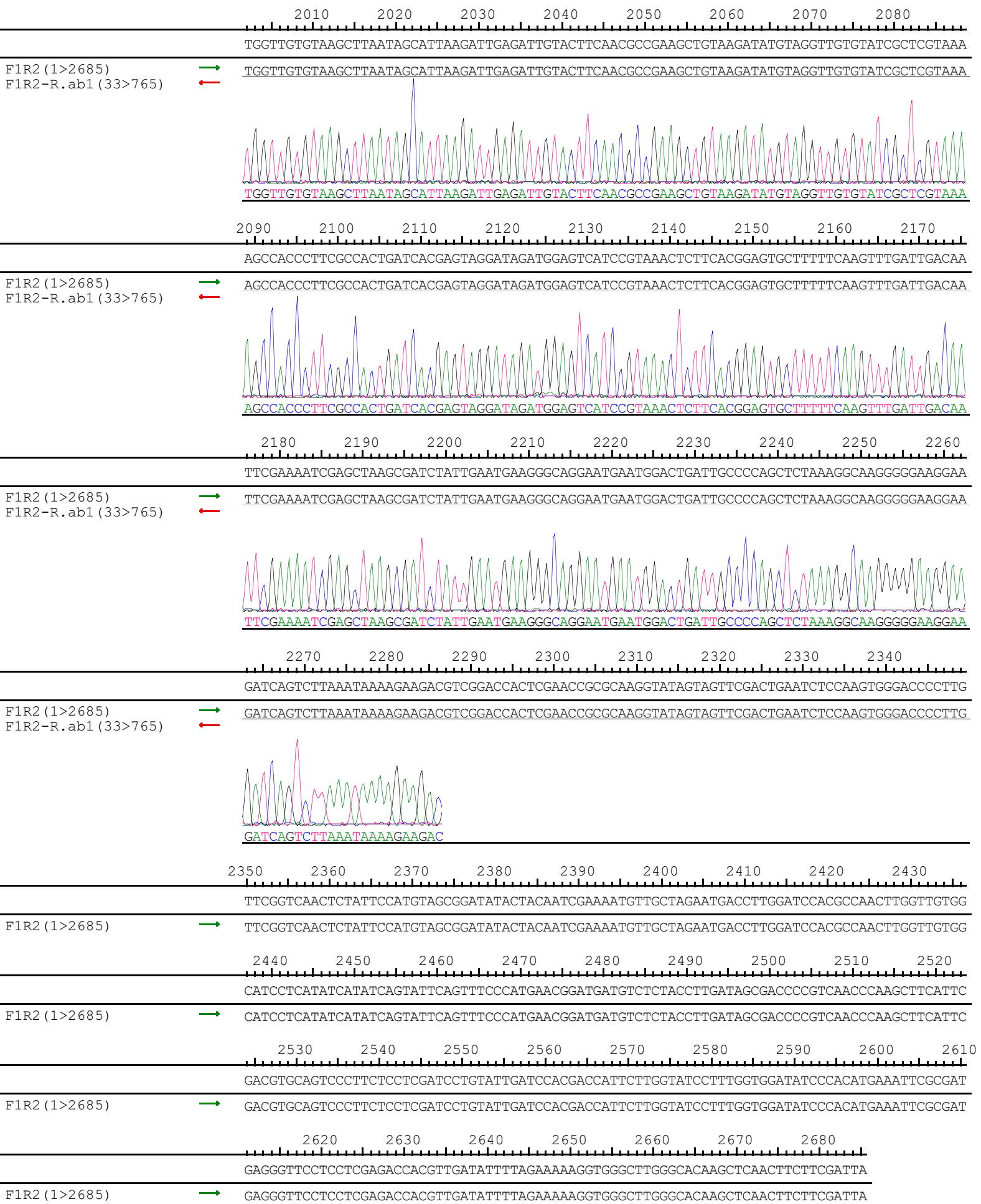

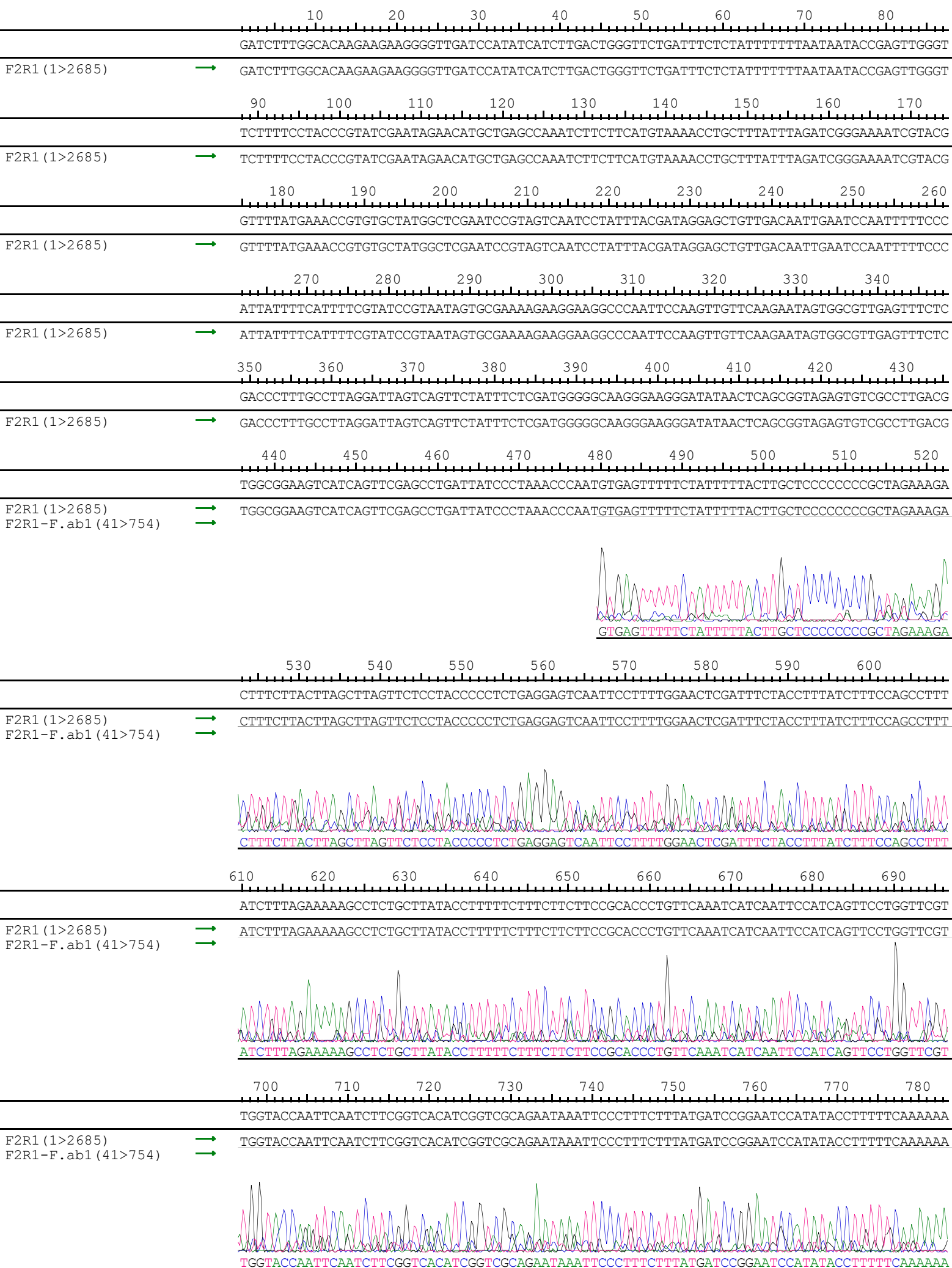

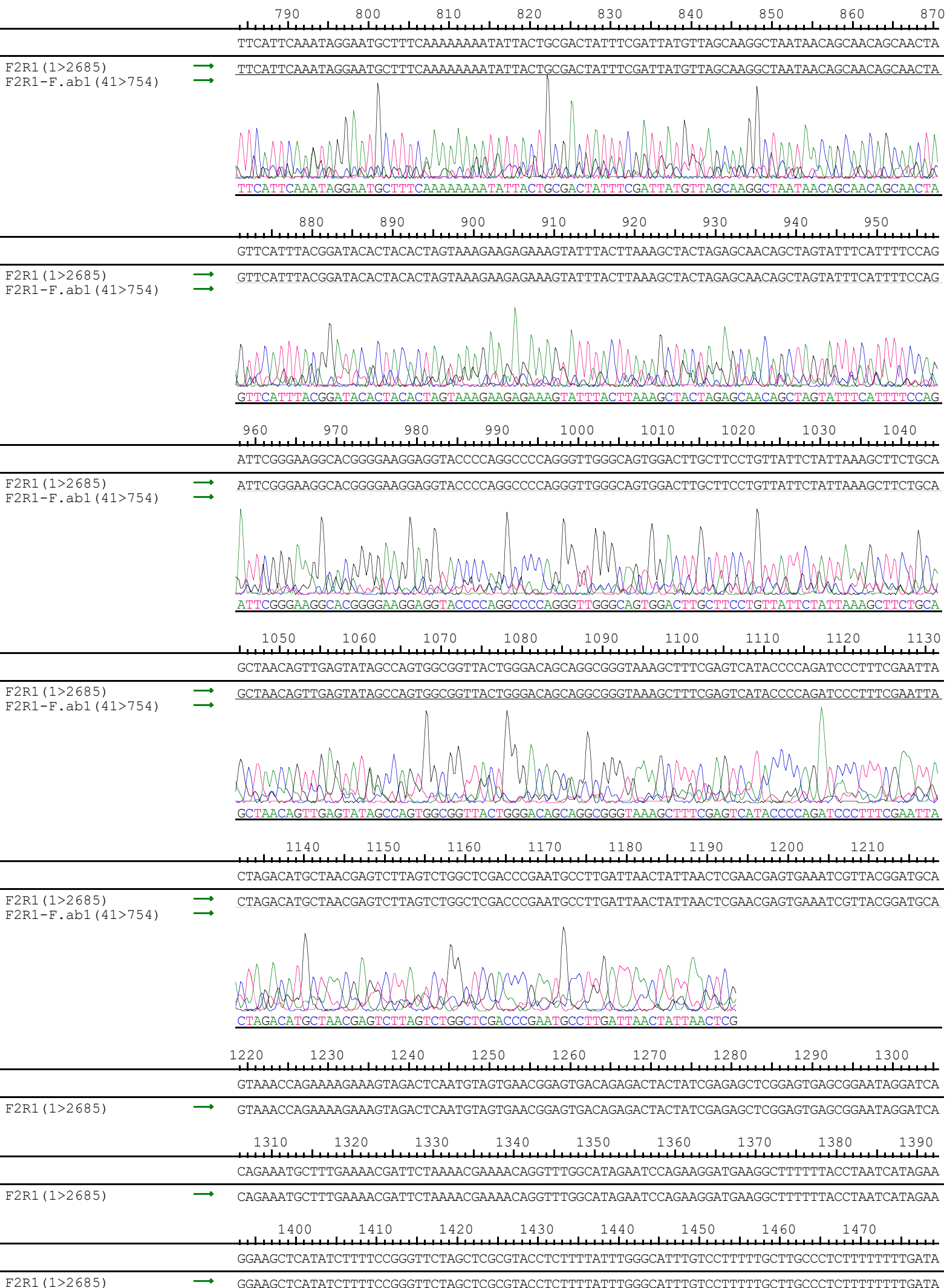

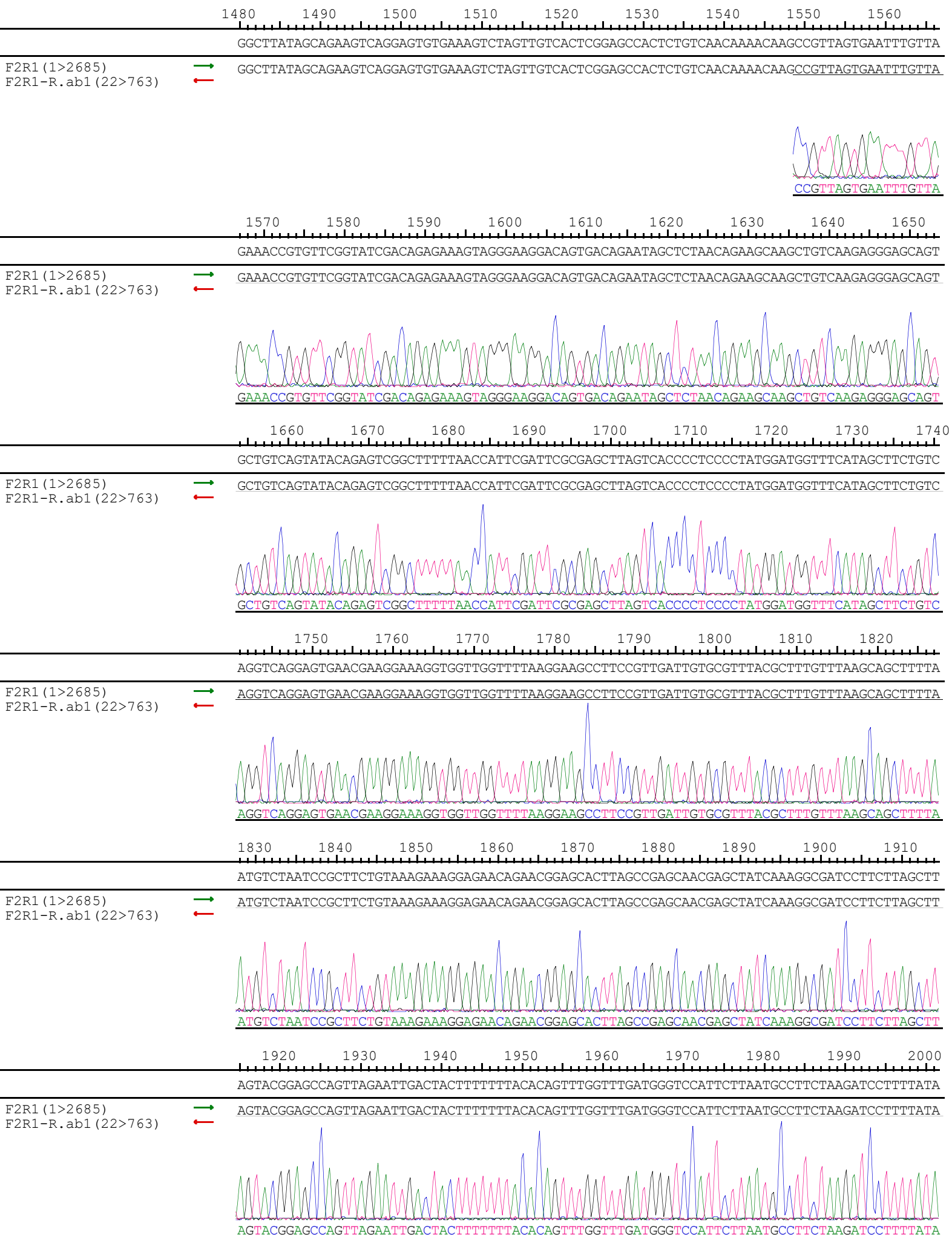

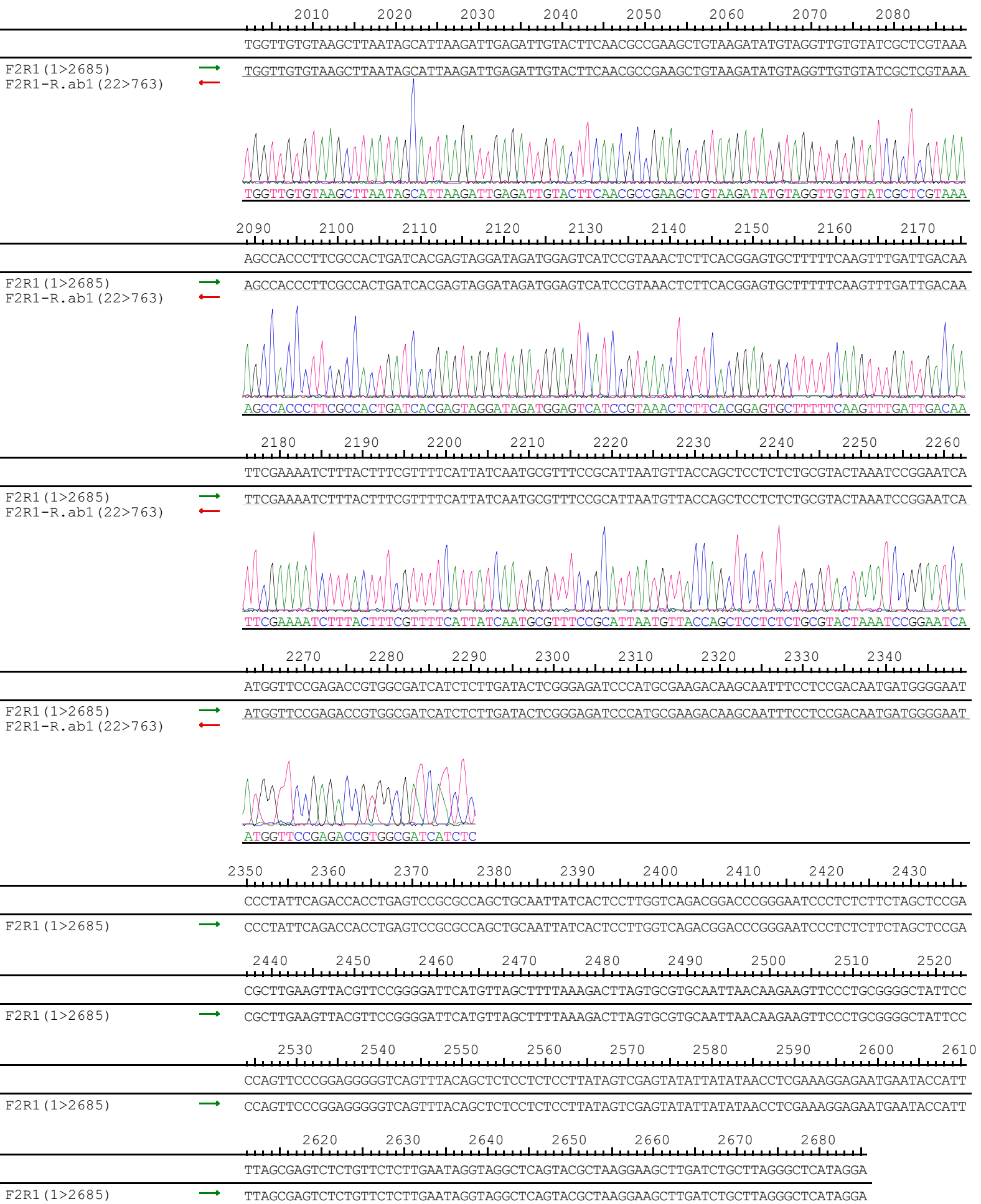

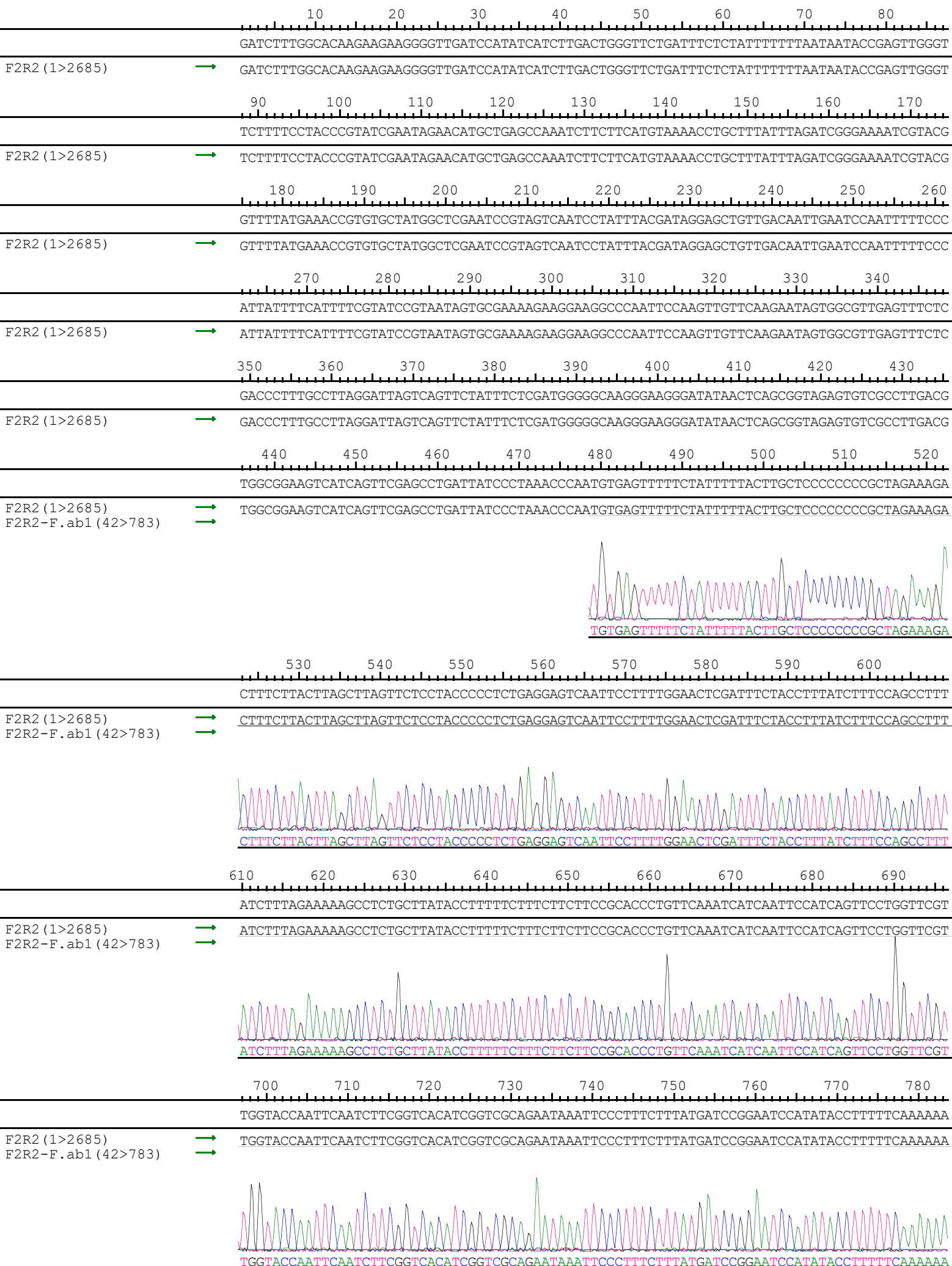



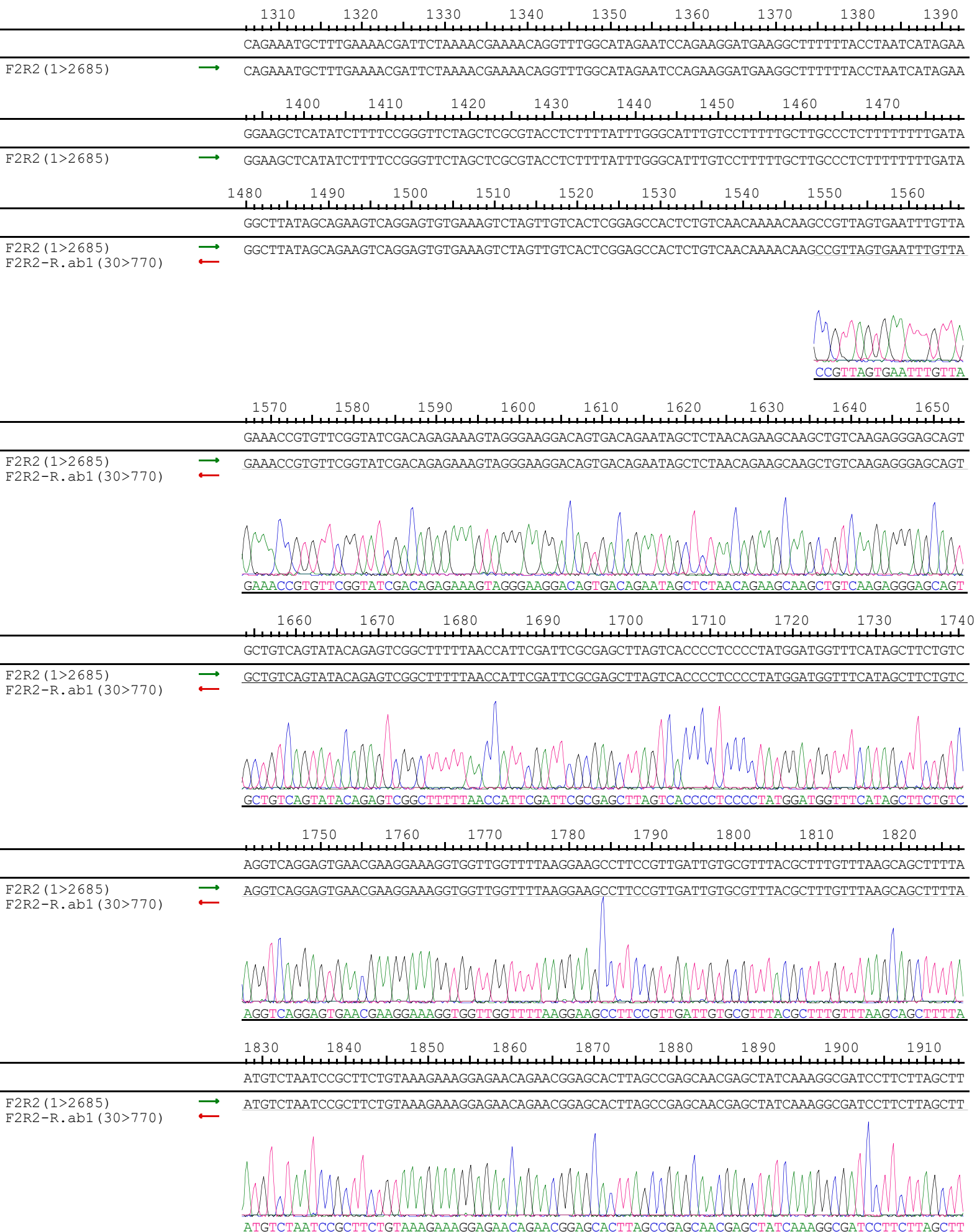

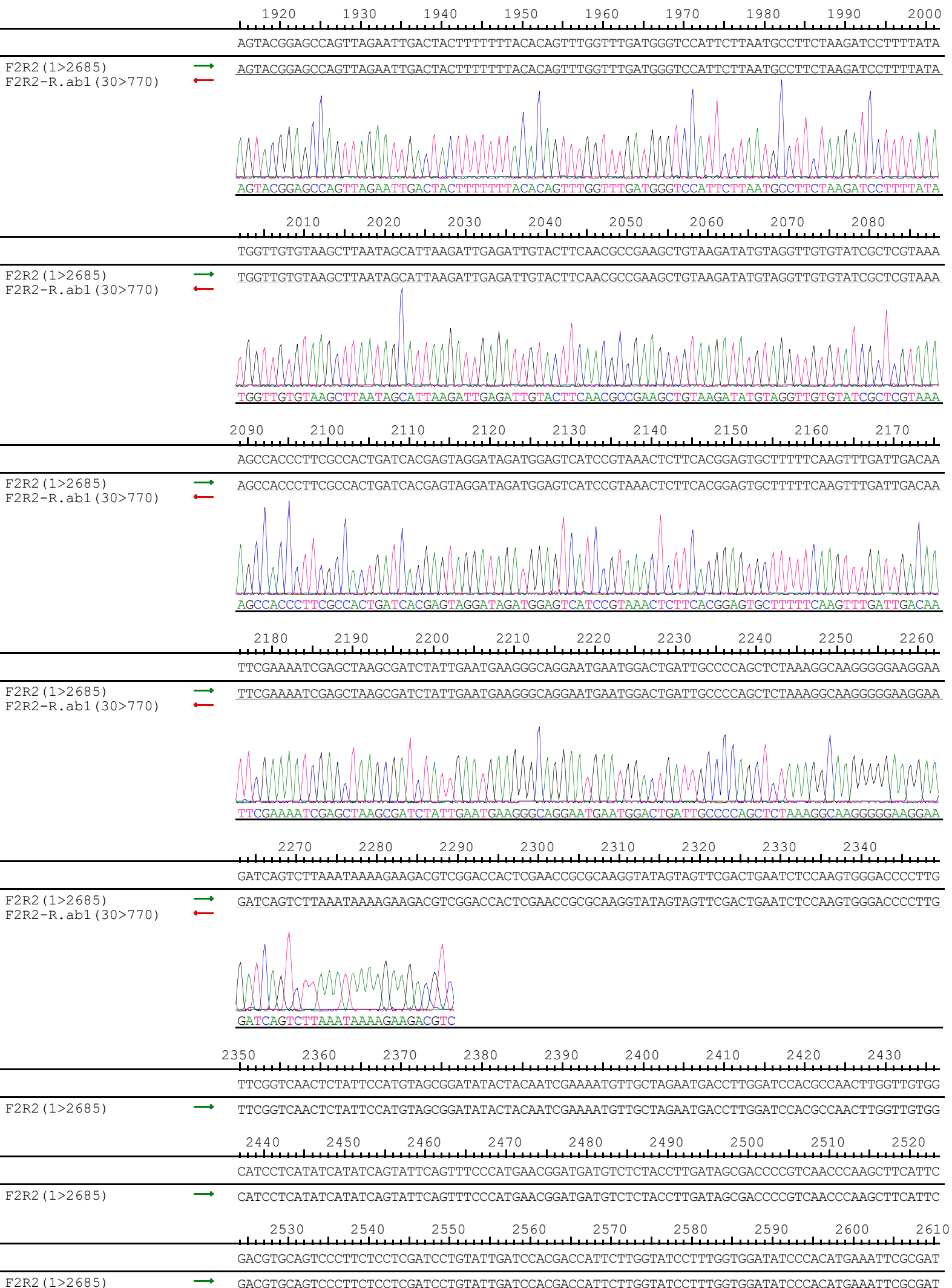

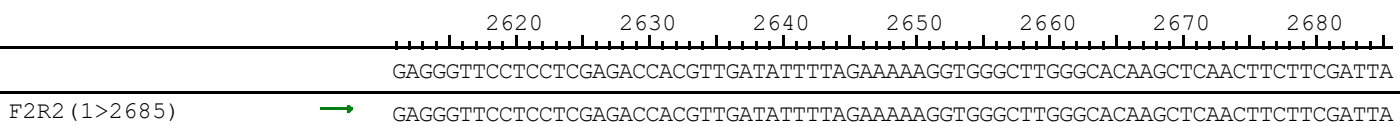

Supplement: Supplementary Figure 4 — Lagenaria siceraria particle MTPT verification. [file DataSheet1.pdf]
